# Neurosteroid Dehydroepiandrosterone Interacts with Nerve Growth Factor (NGF) Receptors, Preventing Neuronal Apoptosis

**DOI:** 10.1371/journal.pbio.1001051

**Published:** 2011-04-26

**Authors:** Iakovos Lazaridis, Ioannis Charalampopoulos, Vassilia-Ismini Alexaki, Nicolaos Avlonitis, Iosif Pediaditakis, Paschalis Efstathopoulos, Theodora Calogeropoulou, Elias Castanas, Achille Gravanis

**Affiliations:** 1 Department of Pharmacology, School of Medicine, University of Crete, Heraklion, Greece; 2 Experimental Endocrinology, School of Medicine, University of Crete, Heraklion, Greece; 3 Institute of Organic and Pharmaceutical Chemistry, National Hellenic Research Foundation, Athens, Greece; The University of Iowa, United States of America

## Abstract

The neurosteroid dehydroepiandrosterone (DHEA), produced by neurons and glia, affects multiple processes in the brain, including neuronal survival and neurogenesis during development and in aging. We provide evidence that DHEA interacts with pro-survival TrkA and pro-death p75^NTR^ membrane receptors of neurotrophin nerve growth factor (NGF), acting as a neurotrophic factor: (1) the anti-apoptotic effects of DHEA were reversed by siRNA against TrkA or by a specific TrkA inhibitor; (2) [^3^H]-DHEA binding assays showed that it bound to membranes isolated from HEK293 cells transfected with the cDNAs of TrkA and p75^NTR^ receptors (K_D_: 7.4±1.75 nM and 5.6±0.55 nM, respectively); (3) immobilized DHEA pulled down recombinant and naturally expressed TrkA and p75^NTR^ receptors; (4) DHEA induced TrkA phosphorylation and NGF receptor-mediated signaling; Shc, Akt, and ERK1/2 kinases down-stream to TrkA receptors and TRAF6, RIP2, and RhoGDI interactors of p75^NTR^ receptors; and (5) DHEA rescued from apoptosis TrkA receptor positive sensory neurons of dorsal root ganglia in NGF null embryos and compensated NGF in rescuing from apoptosis NGF receptor positive sympathetic neurons of embryonic superior cervical ganglia. Phylogenetic findings on the evolution of neurotrophins, their receptors, and CYP17, the enzyme responsible for DHEA biosynthesis, combined with our data support the hypothesis that DHEA served as a phylogenetically ancient neurotrophic factor.

## Author Summary

Dehydroepiandrosterone (DHEA) and its sulphate ester are the most abundant steroid hormones in humans, and DHEA was described as the first neurosteroid produced in the brain. DHEA is known to participate in multiple events in the brain, including neuronal survival and neurogenesis. However, to date no specific cellular receptor has been described for this important neurosteroid. In this study, we provide evidence that DHEA exerts its neurotrophic effects by directly interacting with the TrkA and p75^NTR^ membrane receptors of nerve growth factor (NGF), and efficiently activates their downstream signaling pathways. This activation prevents the apoptotic loss of NGF receptor positive sensory and sympathetic neurons. The interaction of DHEA with NGF receptors may also offer a mechanistic explanation for the multiple actions of DHEA in other peripheral biological systems expressing NGF receptors, such as the immune, reproductive, and cardiovascular systems.

## Introduction

Dehydroepiandrosterone (DHEA) is a steroid, produced in adrenals, in neurons and in glia [1]. The physiological role of brain DHEA appears to be local, i.e. paracrine, while that produced from adrenals, which represents the almost exclusive source of circulating DHEA, is systemic. The precipitous decline of both brain and circulating DHEA with advancing age has been associated with aging-related neurodegenerative diseases [1],[2]. It is experimentally supported that DHEA protects neurons against noxious conditions [3]–[6]. DHEA exerts its multiple pro-survival effects either directly modulating at micromolar concentrations γ-aminobutiric acid type A (GABA_A_), N-methyl-D-aspartate (NMDA), or sigma1 receptors, or following its conversion to estrogens and androgens. We have recently shown that nanomolar concentrations of DHEA protect sympathoadrenal PC12 cells from apoptosis [7]. PC12 cells do not express functional GABA_A_ or NMDA receptors and cannot metabolize DHEA to estrogens and androgens [8]. The anti-apoptotic effect of DHEA in PC12 cells is mediated by high affinity (K_D_ at nanomolar levels) specific membrane binding sites [9]. Activation of DHEA membrane binding sites results in an acute, transient, and sequential phosphorylation of the pro-survival MEK/ERK kinases, which, in turn, activate transcription factors CREB and NFκB, which afford the transcriptional control of anti-apoptotic Bcl-2 proteins. In parallel, activation of DHEA membrane binding sites induces the phosphorylation of PI3K/Akt kinases, leading to phosphorylation/deactivation of the pro-apoptotic Bad protein and protection of PC12 cells from apoptosis [10].

In fact, the anti-apoptotic pathways in sympathoadrenal cells initiated by DHEA at the membrane level strikingly resemble those sensitive to neurotrophin nerve growth factor (NGF). NGF promotes survival and rescues from apoptosis neural crest–derived sympathetic neurons (including their related sympathoadrenal cells) and sensory neurons involved in noniception. NGF binds with high affinity (K_D_: 0.01 nM) to transmembrane tyrosine kinase TrkA receptor and with lower affinity (K_D_: 1.0 nM) to p75^NTR^ receptor, a membrane protein belonging to the TNF receptor superfamily [11]. In the presence of TrkA receptors, p75^NTR^ participates in the formation of high affinity binding sites and enhances NGF responsiveness, leading to cell survival signals. In the absence of TrkA, p75^NTR^ generates cell death signals. Indeed, docking of TrkA by NGF initiates receptor dimerization and phosphorylation of cytoplasmic tyrosine residues 490 and 785 on the receptor. Phosphotyrosine-490 interacts with Shc and other adaptor proteins resulting in activation of PI3K/Akt and MEK/ERK signaling kinase pathways [11]. These signals lead to the activation of prosurvival transcription factors CREB and NFκB, the subsequent production of anti-apoptotic Bcl-2 proteins, and prevention of apoptotic cell death of sympathetic neurons and sympathoadrenal cells, including PC12 cells [12].

Intrigued by the similarities in the prosurvival membrane signaling of DHEA and NGF, we set out to examine in the present study whether the anti-apoptotic effects of DHEA are mediated by NGF receptors. To address this issue we employed a multifaceted approach, designing an array of specific experiments: we used RNA interference (RNAi) to define the involvement of TrkA and p75^NTR^ receptors in the anti-apoptotic action of DHEA; we assessed membrane binding of DHEA in HEK293 cells transfected with the TrkA and p75^NTR^ plasmid cDNAs, using binding assays, confocal laser microscopy, and flow cytometry; to investigate the potential direct physical interaction of DHEA with NGF receptors, we tested the ability of immobilized DHEA to pull-down recombinant or naturally expressed TrkA and p75^NTR^ receptors; finally, we examined the ability of DHEA to rescue from apoptosis NGF receptor sensitive dorsal root ganglia sensory neurons of NGF null mice and NGF deprived rat superior cervical ganglia sympathetic neurons in culture [13]. We provide evidence that DHEA directly binds to NGF receptors to protect neuronal cells against apoptosis, acting as a neurotrophic factor.

## Results

### RNA Interference against TrkA Receptors Reverses the Anti-Apoptotic Effect of DHEA

**Figure 1:**
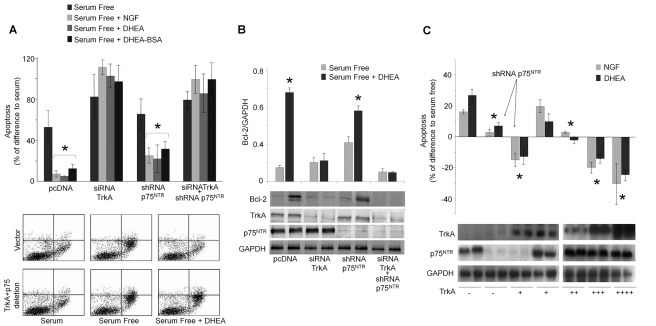
RNA interference against NGF receptors reverses the anti-apoptotic effect of DHEA. PC12 or PC12^nnr5^ cells were transfected with si/shRNAs of TrkA and/or p75^NTR^ (A and B) and/or expressing vectors of TrkA (c). Twenty-four hours later the medium was replaced either with complete medium (serum supplemented) or serum free medium, in the absence or the presence of DHEA, DHEA-BSA (100 nM), or NGF (100 ng/ml). Apoptosis was quantified 24 h later by FACS using Annexin V-FITC and PI. (A) *Upper panel*: levels of apoptosis expressed as % of difference from serum supplemented cells [* *p*<0.01 versus control (serum conditions), *n* = 8]. *Lower panel*: representative FACS analysis of Annexin V-FITC and PI staining. (B) Levels of Bcl-2 protein in serum deprived PC12 cells with or without DHEA treatment. Cellular extracts containing total proteins were collected and levels of Bcl-2 protein were measured by Western blot, and normalized per GAPDH protein content. *Upper panel*: mean ± SE of Bcl-2 levels, normalized against GAPDH (* *p*<0.01 versus control, *n* = 4), *lower panel*: representative Western blots of Bcl-2, TrkA, p75^NTR^, and GAPDH proteins. (C) *Upper panel*: levels of apoptosis in PC12^nnr5^ cells expressed as % of difference from serum deprivation condition. (* *p*<0.01 versus control-naive cells, *n* = 4). *Lower panel*: Western blots of TrkA, p75^NTR^, and GAPDH proteins for each condition.

To test the involvement of NGF receptors in the anti-apoptotic effect of DHEA in serum deprived PC12 cells we have used a combination of three different sequences of siRNAs for TrkA and two different shRNAs for p75^NTR^ transcripts [14]. The effectiveness of si/shRNAs was shown by the remarkable decrease of TrkA and p75^NTR^ protein levels in PC12 cells, observed by immunoblotting analysis, using GAPDH as reference standard (Figure 1B). Scrambled siRNAs were ineffective in decreasing TrkA and p75^NTR^ protein levels and did not significantly alter the effect of DHEA (unpublished data). FACS analysis of apoptotic cells (stained with Annexin V) has shown that DHEA and membrane impermeable DHEA-BSA conjugate at 100 nM diminished the number of apoptotic cells in serum deprived PC12 cell cultures from 53.5%±17.6% increase of apoptosis in serum free condition (control) to 6%±1.4% and 13%±5.2%, respectively (*n* = 8, *p*<0.01 versus control) (Figure 1A). Decreased TrkA expression in serum deprived PC12 cells with siRNAs resulted in the almost complete reversal of the anti-apoptotic effects of NGF and DHEA or DHEA-BSA membrane-impermeable conjugate (Figure 1A). Co-transfection of serum deprived PC12 cells with the si/shRNAs for TrkA and p75^NTR^ receptors did not modify the effect of the TrkA deletion alone. Furthermore, transfection of serum deprived PC12 cells with shRNAs against p75^NTR^ receptor alone did not significantly alter the anti-apoptotic effects of NGF and DHEA, suggesting that their anti-apoptotic effects are primarily afforded by TrkA receptors.

Transfection of serum deprived PC12 cells with the siRNAs against the TrkA transcript fully annulled the ability of DHEA to maintain elevated levels of anti-apoptotic Bcl-2 protein (Figure 1B). Again, transfection with the shRNA against p75^NTR^ receptor alone did not significantly affect Bcl-2 induction by DHEA, further supporting the hypothesis that TrkA is the main mediator of the anti-apoptotic effect of DHEA in this system.

It appears that the ratio of TrkA and p75^NTR^ receptors determines the effect of DHEA or NGF on cell apoptosis and survival. Indeed, both NGF and DHEA induced apoptosis of nnr5 cells, a clone of PC12 cell line, known to express only pro-death p75^NTR^ receptors (Figure 1C), confirming the pro-apoptotic function of this receptor. Blockade of p75^NTR^ expression by shRNA almost completely reversed the pro-apoptotic effect of both agents. The anti-apoptotic effect of NGF and DHEA was remarkably restored after transfection of nnr5 cells with the TrkA cDNA, the efficacy of reversal being proportionally dependent on the amount of transfected TrkA cDNA (Figure 1C).

DHEA was also controlling the response of NGF receptor-positive cells, by regulating TrkA and p75^NTR^ receptor levels, mimicking NGF. Serum deprived PC12 cells were exposed to 100 nM of DHEA or 100 ng/ml of NGF for 12, 24, and 48 h; TrkA and p75^NTR^ protein levels were measured in cell lysates with immunoblotting, using specific antibodies against TrkA and p75^NTR^ proteins, and were normalized against GAPDH. Both NGF and DHEA significantly increased pro-survival TrkA receptor levels in the time frame studied, i.e. from 12 to 48 h (*n* = 5, *p*<0.01) (Figure S1). Furthermore, DHEA and NGF significantly decreased p75^NTR^ receptor levels between 24 and 48 h of exposure (*n* = 5, *p*<0.01).

**Figure 2:**
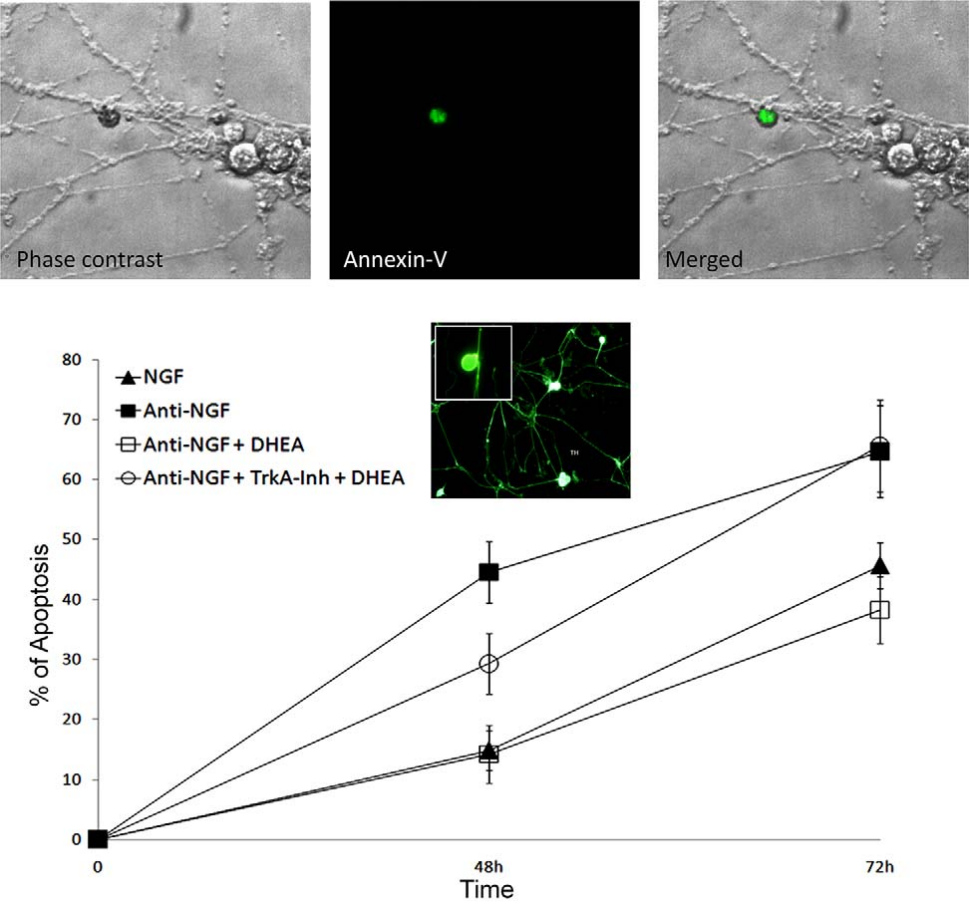
DHEA rescues TrkA positive primary sympathetic neurons from NGF deprivation-induced apoptosis, in a NGF receptor dependent manner. Light and Annexin V-FITC-green stained fluorescence microscopy photographs of dispersed primary sympathetic neurons in culture, isolated from rat superior cervical ganglia (SCG) at P1. SCG dispersed neurons were isolated and cultured for at least 7 d in the presence of the antimitotic drug cytosine-beta-D-arabinofuranoside (AraC) and of NGF before the experiments are performed, in order to obtain an enriched, quasi-homogenous (95%) neuronal cell culture. Sympathetic neurons were cultured in the presence of 100 ng/ml NGF or in the same medium without NGF and containing a polyclonal rabbit anti-NGF-neutralizing antiserum and/or 100 nM DHEA, in the absence or the presence of TrkA-inhibitor. The results shown are the means from three separate experiments where over 300 neurons were counted in six to seven randomly selected optical fields (*p<0.01 versus anti-NGF condition). Inserted photograph depicts tyrosine hydroxylase (TH) staining of sympathetic neurons.

We have also tested the anti-apoptotic effects of DHEA in neural crest deriving superior cervical ganglia (SCG), a classical NGF/TrkA sensitive mammalian neuronal tissue, containing primarily one class of neurons, principal sympathetic neurons. Indeed, NGF and TrkA receptors are absolutely required for SCG sympathetic neuron survival during late embryogenesis and early postnatal development [13],[15]. TrkC receptors are barely detectable after E15.5, and no significant TrkB receptors are present in the SCG at any developmental stage [16]. Dispersed rat sympathetic SCG neurons at P1 were isolated and cultured for at least 7 d in the presence of 100 ng/ml NGF before the experiments are performed, in order to obtain an enriched, quasi-homogenous (95%) neuronal cell culture. Enriched SCGs were then incubated in the presence of 100 ng/ml NGF or in the same medium as above but lacking NGF and containing a polyclonal rabbit anti-NGF-neutralizing antiserum in the absence or the presence of 100 nM DHEA. Withdrawal of NGF strongly increased the number of apoptotic sympathetic neurons stained with Annexin V, while DHEA effectively compensated for NGF by decreasing the levels of apoptotic neurons. This effect was blocked by a specific TrkA inhibitor, thus suggesting the involvement of TrkA receptors as the main mediator of the anti-apoptotic action of DHEA (Figure 2).

### [^3^H]-DHEA Binds to HEK293^TrkA^ and HEK293^p75NTR^ Cell Membranes

We have previously shown the presence of specific DHEA binding sites to membranes isolated from PC12, primary human sympathoadrenal, and primary rat hippocampal cells, with K_D_ at the nanomolar level [9]. The presence of DHEA-specific membrane binding sites on PC12 cells has been confirmed by flow cytometry and confocal laser microscopy of cells stained with the membrane impermeable DHEA-BSA-FITC conjugate. In contrast to estrogens, glucocorticoids and androgens displaced [^3^H]DHEA from its membrane binding sites, acting as pure antagonists by blocking the anti-apoptotic effect of DHEA in serum deprived PC12 cells [9]. In the present study, we repeated this series of experiments using membranes isolated from HEK293 cells transfected with the plasmid cDNAs of TrkA or p75^NTR^ receptors.

**Figure 3:**
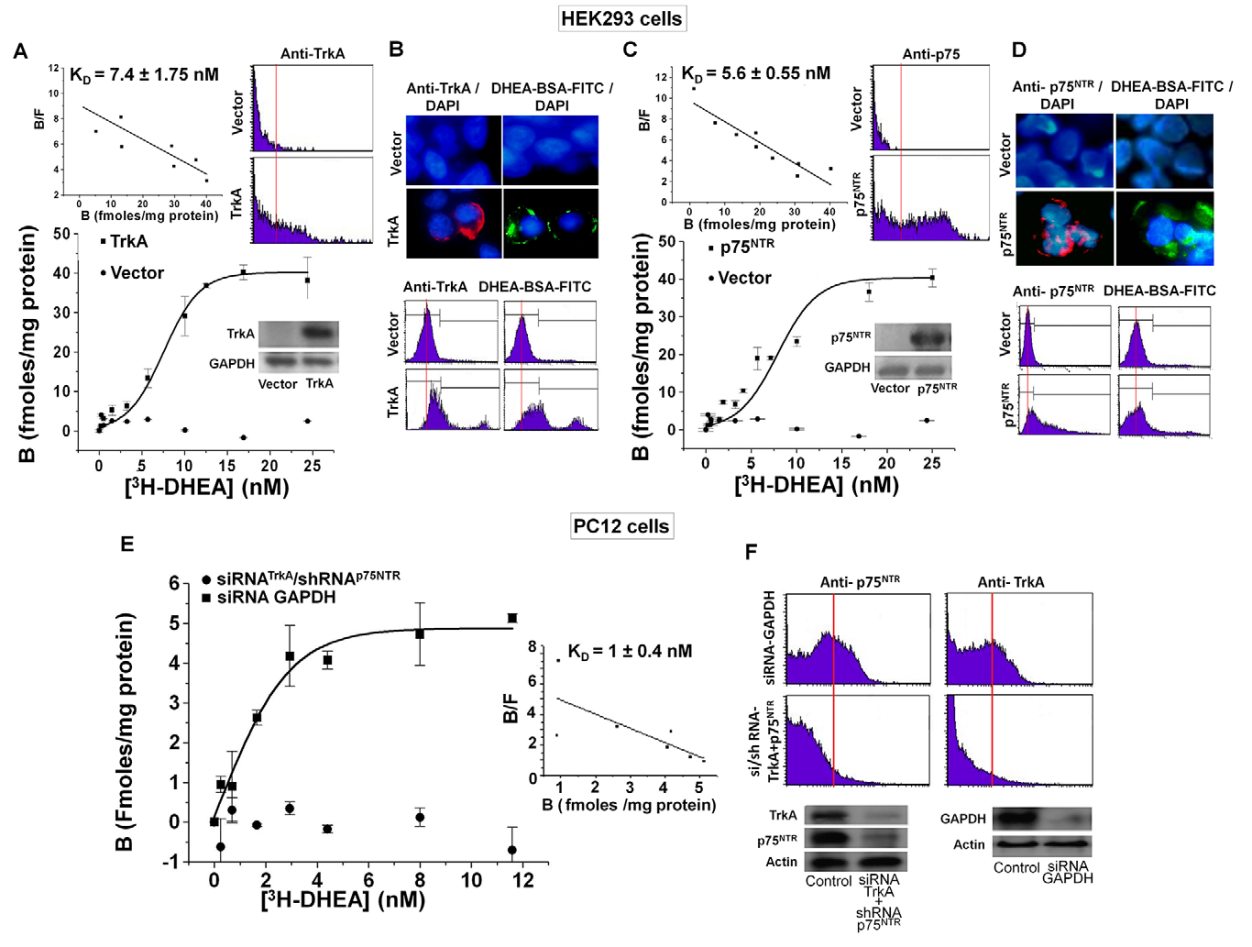
DHEA binds with high affinity to HEK293^TrkA^ and HEK293^p75NTR^ cell membranes. (A, C) [^3^H]-DHEA saturation binding assays and Scatchard blots in HEK293 cells, transfected with the plasmid cDNAs of TrkA and p75^NTR^ receptors. Fifty µl of cell membrane suspension in triplicate were incubated for 30 min at 25°C with 1–30 nM [^3^H]-DHEA in the presence or absence of 500-fold molar excess of DHEA. Western blot inserts show the efficacy of transfection (K_D_ represents the mean ± SE of 3 experiments) (E) [^3^H]-DHEA saturation binding assays in PC12 cells, transfected with the si/shRNAs against TrkA and p75^NTR^ receptors or with the siRNA against GAPDH. The efficacy of transfection is shown in Western blot inserts and in FACS analysis (F), (K_D_ represents the mean ± SE of three experiments). (B, D) Fluorescence localization of DHEA membrane binding on HEK293 cells transfected with the plasmid cDNAs of TrkA and p75^NTR^ receptors. Transfectants were incubated with either the membrane impermeable DHEA-BSA-FITC conjugate (100 nM), BSA-FITC (100 nM), or with specific antibodies against TrkA and p75^NTR^ proteins. Transfectants were analyzed under the confocal laser scanning microscope (B) or by FACS analysis (C). Blue staining depicts Hoechst nuclear staining.

HEK293 cells (not expressing TrkA or p75^NTR^) were transfected with an empty vector (control) or a specific TrkA or p75^NTR^ vector; transfection efficiency was assessed by Western blot (Figure 3A and C,F inserts), confocal laser microscopy, and flow cytometry (Figure 3B,D). Saturation binding experiments have shown that [^3^H]-DHEA bound to membranes isolated from HEK293 cells, transfected with the cDNAs of TrkA or p75^NTR^ receptors. Membranes isolated from HEK293 cells transfected with the empty vector showed no specific binding. The K_D_ values calculated after Scatchard analysis of saturation curves were, for incubation of membranes at 25°C for 30 min, 7.4±1.75 nM and 5.6±0.55 nM for TrkA or p75^NTR^, respectively (*n* = 3) (Figure 3A,C), and for overnight incubation of membranes at 4°C, 7.8±3.1 nM and 5.9±1.7 nM for TrkA or p75^NTR^, respectively (*n* = 3) (Figure S2). DHEA was previously shown to bind with low affinity (K_D_: 2 µM) to androgen receptors (AR) [17]. We have thus tested the hypothesis that specific binding of DHEA to membranes of HEK293 cells transfected with the TrkA and p75^NTR^ cDNAs might be due to the presence of AR receptors, induced by the transfection with NGF receptors. However, RT-PCR analysis showed no detectable levels of androgen receptors mRNA in RNA preparations isolated from naïve and TrkA or p75^NTR^ transfected HEK23 cells (Figure S3).

Transfection of PC12 cells, endogenously expressing NGF receptors, with shRNAs against both TrkA and p75^NTR^ receptors resulted in a complete loss of [^3^H]-DHEA specific membrane binding (Figure 3E,F). To rule out the possibility that the loss of specific binding might be due to the transfection process, we tested binding of [^3^H]-DHEA to membranes isolated from PC12 cells transfected with siRNA against GAPDH. Saturation binding and Scatchard analysis have shown that [^3^H]-DHEA bound to membranes from PC12-siRNA GAPDH cells with a K_D_ = 1.068±0.43 nM (Figure 3E).

The selectivity of DHEA binding to HEK293^TrkA^ and HEK293^p75NTR^ cell membranes was examined by performing heterologous [^3^H]-DHEA displacement experiments using a number of non-labeled steroids or NGF. Binding of [^3^H]-DHEA to membranes isolated from both HEK293^TrkA^ and HEK293^p75NTR^ cells was effectively displaced by NGF (IC_50_: 0.8±0.2 and 1.19±0.45 nM, respectively) (Figure S4). NGF was also effective in displacing [^3^H]-DHEA binding on membranes isolated from PC12 cells (IC_50_: 0.92±0.32 nM, unpublished data). Estradiol failed to displace [^3^H]-DHEA from its binding to membranes from HEK293^TrkA^ and HEK293^p75NTR^ cells at concentrations ranging from 0.1 to 1000 nM. In contrast, displacement of [^3^H]-DHEA binding to membranes from both HEK293^TrkA^ and HEK293^p75NTR^ cells was shown by sulfated ester of DHEA, DHEAS (IC_50_: 6.1±1.1 and 8.1±1.2 nM, respectively, *n* = 3), and testosterone (Testo) (IC_50_: 5.3±2.1 and 7.4±3.2 nM, respectively). Glucocorticoid dexamethasone (DEX) effectively competed [^3^H]-DHEA binding to membranes from HEK293^TrkA^ (IC_50_: 9.5±4.6 nM) but was ineffective in displacing DHEA binding to membranes from HEK293^p75NTR^ cells. Homologous [^125^I]-NGF displacement experiments with unlabeled NGF confirmed the presence of specific NGF binding on membranes from both HEK293^TrkA^ and HEK293^p75NTR^ cells with IC_50_ 0.3±0.09 and 1.7±0.38 nM, respectively. It is of note that in contrast to unlabeled NGF, DHEA was unable to displace binding of [^125^I]-NGF to membranes isolated from HEK293^TrkA^ and HEK293^p75NTR^ transfectants (unpublished data).

### DHEA-BSA-FITC Conjugate Stains HEK293^TrkA^ and HEK293^p75NTR^ Cell Membranes

Incubation of PC12 cells with the membrane impermeable, fluorescent DHEA-BSA-fluorescein conjugate results in a specific spot-like membrane fluorescent staining [9]. In the present study, we have tested the ability of DHEA-BSA-FITC conjugate to stain HEK293^TrkA^ and HEK293^p75NTR^ transfectants. Fluorescence microscopy analysis revealed that DHEA-BSA-FITC clearly stained the membranes of HEK293^TrkA^ and HEK293^p75NTR^ cells (Figure 3B,D). No such staining was found in non-transfected HEK293 cells (unpublished data) or in HEK293 cells transfected with the vectors empty of TrkA and p75^NTR^ cDNAs (Figure 3B,D). Furthermore, BSA-FITC conjugate was ineffective in staining both transfectants (unpublished data). We have further confirmed the presence of membrane DHEA-BSA-FITC staining of HEK293^TrkA^ and HEK293^p75NTR^ cells with flow cytometry (FACS) analysis (Figure 3B,D). Specific staining was noted in both transfectants. No such staining was seen in non-transfected HEK293 cells (unpublished data) or in HEK293 cells transfected with the empty vectors (Figure 3B,D). In both fluorescence microscopy and FACS experiments membrane staining of TrkA or p75^NTR^ proteins in HEK293^TrkA^ and HEK293^p75NTR^ cells was also shown using specific antibodies for each protein (Figure 3B,D).

### Immobilised DHEA Pulls Down TrkA and p75^NTR^ Receptors

**Figure 4:**
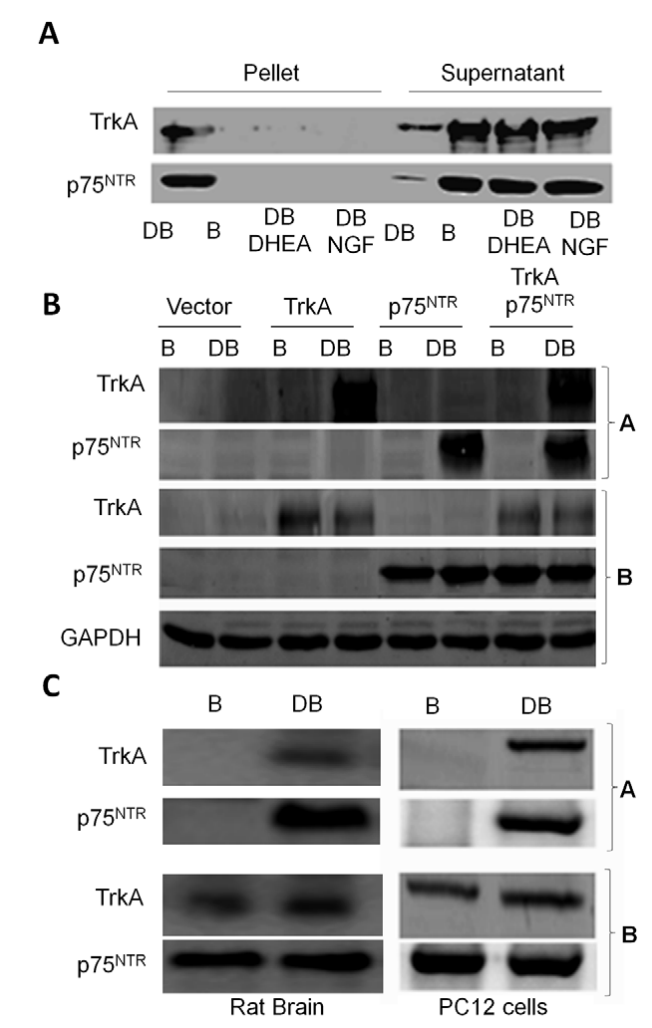
Immobilised DHEA pulls down TrkA and p75^NTR^ receptors. Covalently linked DHEA-7-*O*-(carboxymethyl) oxime (DHEA-7-CMO) to polyethylene glycol amino resin (NovaPEG amino resin) was incubated with recombinant TrkA and p75^NTR^ proteins (A) or with cell extracts isolated from HEK293^TrkA^ HEK293^p75NTR^ transfectants (B), PC12 cells, and whole rat brain (C). Precipitation experiments (panels marked with A show Western blot analysis of precipitates while panels marked with B show Western blot analysis of total lysates) with specific antibodies against TrkA and p75^NTR^ proteins were performed as described in Materials and Methods. DB, DHEA-7-*O*-(carboxymethyl) oxime (DHEA-7-CMO) polyethylene glycol amino resin; B: polyethylene glycol amino resin, DB-DHEA, DB-NGF pre-incubation of the recombinant proteins with DHEA or NGF, respectively; P, pellet; S, supernatant.

Our binding assays with radiolabeled DHEA suggest that DHEA physically interacts with NGF receptors. To test this hypothesis we covalently linked DHEA-7-*O*-(carboxymethyl) oxime (DHEA-7-CMO) to polyethylene glycol amino resin (NovaPEG amino resin) and tested the ability of immobilized DHEA to pull down TrkA and p75^NTR^ proteins. Precipitation experiments and Western blot analysis of precipitates with specific antibodies against TrkA and p75^NTR^ proteins (Figure 4A) showed that immobilized DHEA effectively precipitated recombinant TrkA and p75^NTR^ proteins, while pre-incubation of the recombinant proteins with DHEA or NGF in excess abolished the ability of DHEA-PEG to pull down both receptors. Similar results were obtained when cell extracts isolated from HEK293 cells transfected with TrkA and p75^NTR^ cDNAs, PC12 cells, and whole rat brain were treated with immobilized DHEA (Figure 4B, panels marked with A). No precipitation of TrkA and p75^NTR^ proteins was shown with polymer-supported DHEA-7-CMO incubated with cell extracts from untransfected HEK293 cells or HEK293 cells transfected with the empty vectors. A control experiment was performed with NovaPeg amino resin (no DHEA-7-CMO present), which was found ineffective in precipitating TrkA and p75^NTR^ proteins (Figure 4). The presence of TrkA and p75^NTR^ receptors in HEK293^TrkA^ and HEK293^p75NTR^ transfectants and in PC12 and fresh rat brain was confirmed with Western blot analysis using specific antibodies against TrkA and p75^NTR^ proteins and GAPDH as reference standard (Figure 4, panels marked with B).

### DHEA Induces TrkA- and p75^NTR^-Mediated Signaling

**Figure 5:**
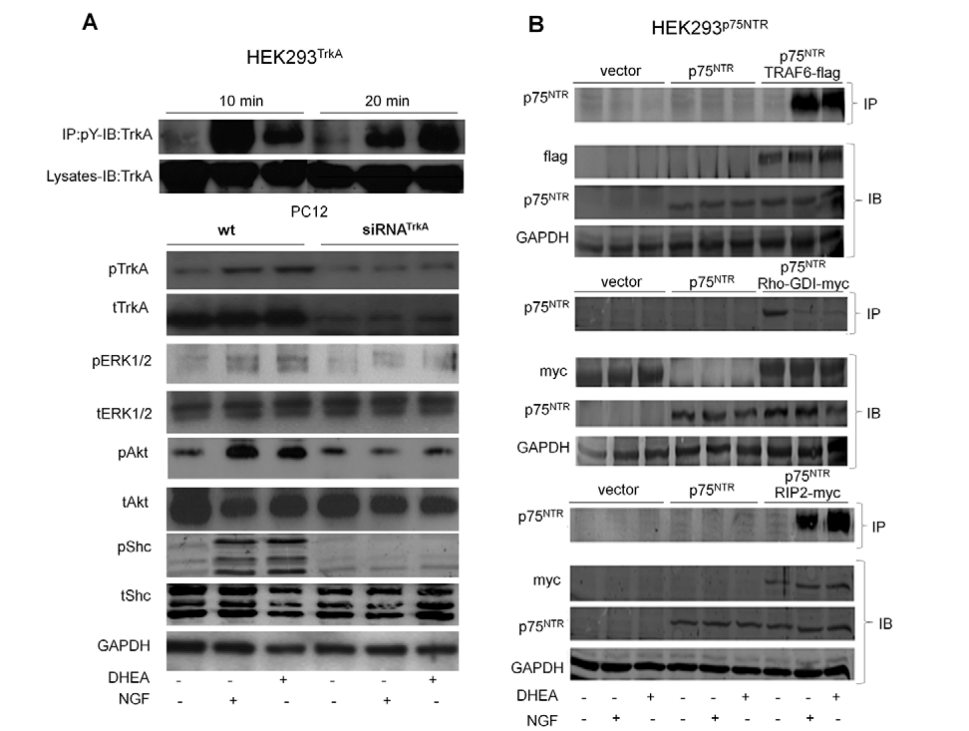
DHEA induces TrkA- and p75^NTR^-mediated signaling. (A) *upper panel*: HEK293^TrkA^ transfectants were exposed for 10 and 20 min to 100 nM of DHEA or 100 ng/ml of NGF, and cell lysates were immunoprecipitated with anti-tyrosine antibodies and analyzed by Western blotting, using specific antibodies against TrkA receptors. *lower panel*: Serum deprived naive or siRNA^TrkA^ transfected PC12 cells were incubated for 10 min with 100 nM of DHEA or 100 ng/ml of NGF and cell lysates were analyzed with Western blotting, using specific antibodies against the phosphorylated and total forms of TrkA receptor and of Shc, ERK1/2, and Akt kinases. (B) HEK293 cells were co-transfected with the plasmid cDNAs of p75^NTR^ and of each one of the effectors TRAF6, RIP2, or RhoGDI, tagged with the flag (TRAF6) or myc (RIP2, RhoGDI) epitopes. Transfectants were exposed for 30 min to 100 nM of DHEA or 100 ng/ml of NGF, and lysates were immunoprecipitated with antibodies against flag or myc, followed by immunoblotting with p75^NTR^ specific antibodies.

Previous findings have shown that NGF controls the responsiveness of sensitive cells through induction of TrkA phosphorylation and regulation of the levels of each one's receptors [18]. We compared the ability of NGF and DHEA to induce phosphorylation of TrkA in HEK293 cells transfected with the cDNAs of TrkA receptors. HEK293^TrkA^ transfectants were exposed for 10 and 20 min to 100 nM of DHEA or 100 ng/ml of NGF, and cell lysates were immunoprecipitated with anti-tyrosine antibodies and analyzed by Western blotting, using specific antibodies against TrkA receptors. Both NGF and DHEA strongly increased phosphorylation of TrkA as early as 10 min, an effect which was also maintained at 20 min (Figure 5A). We also tested the effects of DHEA and NGF in PC12 cells, endogenously expressing TrkA receptors. Naive or siRNA^TrkA^ transfected PC12 cells were incubated for 10 min with DHEA or NGF, and cell lysates were analyzed with Western blotting, using specific antibodies against Tyr490-phosphorylated TrkA and total TrkA. Both NGF and DHEA strongly induced the phosphorylation of TrkA in naive PC12 cells, effects which were diminished in siRNA^TrkA^ transfected PC12 cells (Figure 5A). The stimulatory effect of DHEA on TrkA phosphorylation might be due to an increase of NGF production. To test this hypothesis, we measured with ELISA the levels of NGF in culture media of HEK293 and PC12 cells exposed for 5 to 30 min to 100 nM of DHEA. NGF levels in culture media of control and DHEA-treated HEK293 and PC12 cells were undetectable, indicating that DHEA-induced TrkA phosphorylation was independent of NGF production. DHEAS mimicked the effect of DHEA and rapidly induced (within 10 min) the phosphorylation of TrkA receptors in HEK293 transfected with the TrkA cDNA expression vector (Figure S5). On the other hand, testosterone, while capable of displacing DHEA binding to TrkA receptors, was unable to increase phosphorylation of TrkA in the same system (Figure S5).

We compared the ability of NGF and DHEA to induce phosphorylation of TrkA-sensitive Shc, ERK1/2, and Akt kinases. Serum deprived naive or siRNA^TrkA^ transfected PC12 cells were incubated for 10 min with 100 nM DHEA or 100 ng/ml NGF and cell lysates were analyzed with Western blotting, using specific antibodies against the phosphorylated and total forms of kinases mentioned above. Both DHEA and NGF strongly increased phosphorylation of Shc, ERK1/2, and Akt kinases in naive PC12 cells, effects which were almost absent in siRNA^TrkA^ transfected PC12 cells, suggesting that both DHEA and NGF induce Shc, ERK1/2, and Akt phosphorylation via TrkA receptors (Figure 5A).

The effectiveness of DHEA to promote the interaction of p75^NTR^ receptors with its effector proteins TRAF6, RIP2, and RhoGDI was also assessed. It is well established that NGF induces the association of p75^NTR^ receptors with TNF receptor-associated factor 6 (TRAF6), thus facilitating nuclear translocation of transcription factor NFκB [19]. Furthermore, p75^NTR^ receptors associate with receptor-interacting protein 2 (RIP2) in a NGF-dependent manner [20]. RIP2 binds to the death domain of p75^NTR^ via its caspase recruitment domain (CARD), conferring nuclear translocation of NFκB. Finally, naive p75^NTR^ interacts with RhoGDP dissociation inhibitor (RhoGDI), activating small GTPase RhoA [21]. In that case, NGF binding abolishes the interaction of p75^NTR^ receptors with RhoGDI, thus inactivating RhoA. We co-transfected HEK293 cells with the plasmid cDNAs of p75^NTR^ and of each one of the effectors TRAF6, RIP2, or RhoGDI, tagged with the flag (TRAF6) or myc (RIP2, RhoGDI) epitopes. Transfectants were exposed to 100 nM DHEA or 100 ng/ml NGF, and lysates were immunoprecipitated with antibodies against flag or myc, followed by immunoblotting with p75^NTR^ specific antibodies. Both DHEA and NGF efficiently induced the association of p75^NTR^ with effectors TRAF6 and RIP2, while facilitating the dissociation of RhoGDI from p75^NTR^ receptors (Figure 5B).

### DHEA Reverses the Apoptotic Loss of TrkA Positive Sensory Neurons in Dorsal Root Ganglia of NGF Null Mouse Embryos

**Figure 6:**
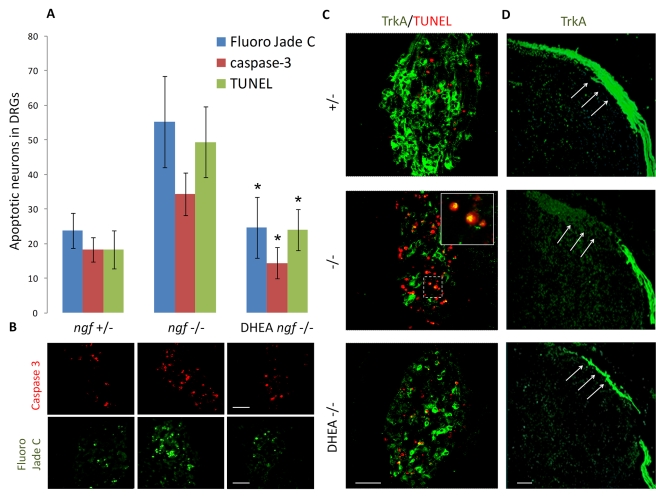
DHEA decreases the apoptotic loss of TrkA positive sensory neurons in dorsal root ganglia of NGF null mouse embryos. Heterozygous mice for the NGF deletion were interbred to obtain mice homozygous for the NGF gene disruption. The mothers were treated daily with an intraperitoneal injection of DHEA (2 mg) or vehicle (4.5% ethanol in 0.9% saline). Embryos were collected at E14 day of pregnancy as described in Material and Methods, and sections were stained for various apoptotic and neuronal markers: (A) Caspase 3, Fluoro Jade C,and TUNEL positive neurons were counted. The results shown are the mean ± SE from three embryos in each group. In each embryo apoptotic neurons were counted in at least eight sections from different DRGs (* *p*<0.01 versus NGF null mice). (B) Staining for Caspase 3 and Fluoro jade C, markers of apoptotic and degenerative neurons, respectively; (C) double staining for TrkA positive and TUNEL apoptotic neurons (note the co-staining of apoptotic neurons for both TrkA and TUNNEL in NGF null embryos); and (D) TrkA positive collaterals of DRG sensory neurons. Scale bars at B, C, and D: 200 µm.

NGF null mice have fewer sensory neurons in dorsal root ganglia (DRG) due to their apoptotic loss [13]. Heterozygous mice for the NGF deletion were interbred to obtain mice homozygous for the NGF gene disruption. The mothers were treated daily with an intraperitoneal injection of DHEA (2 mg) or vehicle (4.5% ethanol in 0.9% saline). Embryos were collected at E14 day of pregnancy and sections were stained for Caspase 3 and Fluoro jade C, markers of apoptotic and degenerative neurons, respectively. *ngf*−*/*− embryos at E14 showed a dramatic increase in the number of Fluoro Jade C and Caspase 3 positive neurons in the DRG compared to the *ngf+/*− embryos (Figure 6A,B). DHEA treatment significantly reduced Fluoro Jade C and Caspase 3 positive neurons in the DRG to levels of *ngf+/*− embryos. Furthermore, TrkA and TUNEL double staining of DRGs has shown that in *ngf+/*− embryos, numbers of TUNEL-positive apoptotic neurons were minimal, while TrkA positive staining was present in a large number of neuronal cell bodies of the DRG and their collaterals were extended within the marginal zone to the most dorsomedial region of the spinal cord. On the contrary, in DRG of *ngf*−*/*− embryos levels of TUNEL-positive apoptotic neurons were dramatically increased, while TrkA neuronal staining was considerably decreased and DRG collaterals of the dorsal funiculus were restricted in the dorsal root entry zone (Figure 6C). DHEA treatment resulted in a significant increase of TrkA positive staining and the extension of TrkA staining within the marginal zone to the most dorsomedial region of the spinal cord similarly to the *ngf+/*− embryos (Figure 6D), while staining of TUNEL-positive apoptotic neurons was decreased to levels shown in *ngf+/*− embryos.

## Discussion

DHEA exerts multiple actions in the central and peripheral nervous system; however, no specific receptor has been reported to date for this neurosteroid. Most of its actions in the nervous tissue were shown to be mediated via modulation, at micromolar concentrations, of membrane neurotransmitter receptors, such as NMDA, GABA_A_, and sigma1 receptors. DHEA may also influence brain function by direct binding, also at micromolar concentrations, to dendritic brain microtubule-associated protein MAP2C [22]. In the present study we provide evidence that DHEA binds to NGF receptors. This is the first report showing a direct binding of a steroid to neurotrophin receptors. Saturation experiments and Scatchard analysis of [^3^H]-DHEA binding to membranes isolated from HEK293 cells transfected with the cDNAs of TrkA and p75^NTR^ receptors showed that DHEA binds to both membranes (7.4±1.75 nM and 5.6±0.55 nM for TrkA or p75^NTR^, respectively). Non-radioactive NGF effectively displaced [^3^H]-DHEA binding to both membrane preparations, with IC_50_: 0.8±0.2 and 1.19±0.45 nM, respectively. Furthermore, pull-down experiments using DHEA covalently immobilized on NovaPEG amino resin suggest that DHEA binds directly to TrkA and p75^NTR^ proteins. Indeed, polymer-supported DHEA-7-CMO effectively pulled down recombinant TrkA and p75^NTR^ proteins and precipitated both proteins from extracts prepared from cells expressing both receptors (HEK293^TrkA^, HEK293^p75NTR^, and PC12 cells and freshly isolated rat brain). Interestingly, DHEA was unable to effectively displace binding of [^125^I]-NGF on membranes isolated from HEK293^TrkA^ and HEK293^p75NTR^ transfectants. It is possible that dissociation of binding of peptidic NGF from its receptors lasts longer due to the multiple sites of interaction within the binding cleft of this large peptidic molecule compared to smaller in volume steroid. Another explanation might be that NGF and DHEA bind to different domains of NGF receptors, the NGF domain being non-recognizable by DHEA. It is of note that antidepressant amitryptiline cannot chase NGF from TrkA receptors because it binds to a different domain on TrkA protein compared to NGF. Indeed, other small molecules, like antidepressant amitriptyline and gamboge's natural extract gambogic amide, bind in the extracellular and the cytoplasmic juxtamembrane domains of TrkA receptor, although with much lower affinity compared to DHEA (K_d_ 3 µM and 75 nM, respectively) [23],[24]. The domains of TrkA and p75^NTR^ proteins involved in DHEA binding were not defined in the present study. Mutagenesis assays combined with NMR spectroscopy are planned to map the domains of both receptors related to DHEA binding.

Our findings suggest that binding of DHEA to NGF receptors is functional, mediating its anti-apoptotic effects. Indeed, blocking of TrkA expression by RNAi almost completely reversed the ability of DHEA to protect PC12 cells from serum deprivation-induced apoptosis and to maintain elevated levels of the anti-apoptotic Bcl-2 protein. Additionally, in dispersed primary sympathetic neurons in culture, DHEA effectively compensated NGF deprivation by decreasing the levels of apoptotic neurons, an effect which was reversed by a specific TrkA inhibitor, further supporting the involvement of TrkA receptors in the anti-apoptotic action of DHEA. Finally, DHEA effectively rescued from apoptosis TrkA-positive dorsal root ganglia sensory neurons of NGF null mouse embryos.

It appears that the decision between survival and death among DHEA-responsive cells is determined by the ratio of TrkA and p75^NTR^ receptors. In fact, DHEA and NGF induced apoptosis of nnr5 cells, a clone of PC12 cells expressing only pro-death p75^NTR^ receptors. The pro-death effects of both agents were completely blocked by p75^NTR^ shRNA and were remarkably restored after transfection of nnr5 cells with the TrkA cDNA. It is of note that during brain development the ratio of TrkA to p75^NTR^ varies tempospatially [25]. Thus, the ability of DHEA to act in a positive or negative manner on neuronal cell survival may depend upon the levels of the two receptors during different stages of neuronal development.

Binding of DHEA on both TrkA and p75^NTR^ receptors was effectively competed by sulfated DHEA, DHEAS (IC_50_: 6.1±1.1 and 8.1±1.2 nM, respectively), suggesting that DHEAS may also bind to NGF receptors. Testosterone displaced DHEA binding to TrkA and p75^NTR^ (IC_50_: 5.3±2.1 and 7.4±3.2 nM, respectively), while synthetic glucocorticoid dexamethasone displaced DHEA binding only to pro-survival TrkA receptors (IC_50_: 9.5±4.6 nM). In a previous study we had shown that both steroids effectively displaced DHEA from its specific membrane binding sites of sympathoadrenal cells, acting as DHEA antagonists by blocking its anti-apoptotic effect and the induction of anti-apoptotic Bcl-2 proteins [9]. Our findings suggest that testosterone and glucocorticoids may act as neurotoxic factors by antagonizing endogenous DHEA and NGF for their binding to NGF receptors, explaining previously published data. Indeed, testosterone was shown to increase NMDA and GABA_A_-mediated neurotoxicity [26],[27]. Our findings suggest that testosterone may act as a neurotoxic factor by also antagonizing the neuroprotective effects of endogenous DHEA. Furthermore, glucocorticoids show a bimodal effect on hippocampal neurons causing acutely an increase in performance of spatial memory tasks, while chronic exposure has been associated with decreased cognitive performance and neuronal atrophy [28]. Acute administration of glucocorticoids results in a glucocorticoid receptor-mediated phosphorylation and activation of hippocampal TrkB receptors, exerting trophic effects on dentate gyrus hippocampal neurons [29], via an increase in the sensitivity of hippocampal cells to neurotrophin BDNF, the endogenous TrkB ligand known to promote memory and learning [30]. However, overexposure to glucocorticoids during prolonged periods of stress is detrimental to central nervous system neurons, especially in aged animals, affecting mainly the hippocampus. It is possible that part of neurotoxic effects of glucocorticoids may be due to their antagonistic effect on the neuroprotective effect of endogenous DHEA and NGF, via TrkA receptor antagonism. The decline of brain DHEA and NGF levels during aging and in Alzheimer's disease [28] might exacerbate this phenomenon, rendering neurons more vulnerable to glucocorticoid toxicity. Indeed, glucocorticoid neurotoxicity becomes more pronounced in aged subjects since cortisol levels in the cerebrospinal fluid increase in the course of normal aging, as well as in relatively early stages of Alzheimer's disease [28].

A number of neurodegenerative conditions are associated with lower production or action of both DHEA and NGF [31],[32]. Animal studies suggest that NGF may reverse, or slow down the progression of Alzheimer's related cholinergic basal forebrain atrophy [32]. Furthermore, the neurotrophic effects of NGF in experimental animal models of neurodegenerative conditions, like MPTP (Parkinson's disease), experimental allergic encephalomyelitis (multiple sclerosis), or ischemic retina degeneration mice [33]–[35] support its potential as a promising neuroprotective agent. However, the use of NGF in the treatment of these conditions is limited, because of its poor brain blood barrier permeability. It is of interest that DHEA also exerts neuroprotective properties in some of these animal models [7],[36]. These findings suggest that synthetic DHEA analogs, deprived of endocrine effects, may represent a new class of brain blood barrier permeable NGF receptor agonists with neuroprotective properties. We have recently reported the synthesis of 17-spiro-analogs of DHEA, with strong anti-apoptotic and neuroprotective properties, deprived of endocrine effects [37], which are now being tested for their ability to bind and activate NGF receptors.

**Figure 7:**
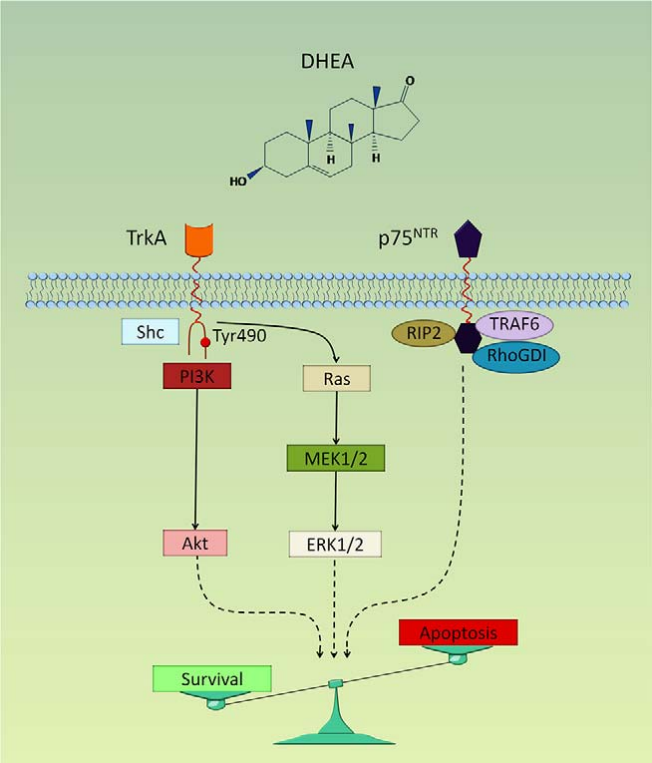
Hypothetical model of NGF receptor-mediated signaling pathways involved in the effects of DHEA on neuronal cell fate. DHEA binds with high affinity to TrkA and p75^NTR^ receptors, initiating the following sequence of events: 1) DHEA induces TrkA-mediated Tyr490-phosphorylation of Shc, ERK1/2, and Akt kinases, controlling the expression and function of apoptotic Bcl-2 proteins; and 2) DHEA promotes the interaction of p75^NTR^ receptors with effector proteins TRAF6, RIP2, and RhoGDI affecting neuronal cell apoptosis. Our findings suggest that the decision between survival and death among DHEA-responsive cells is determined by the balance of its interactions with TrkA and p75^NTR^ receptors.

We have previously defined the pro-survival signaling pathways that are initiated by DHEA at the membrane level [3]. These pathways include MEK1/2/ERK1/2 and PI3K/Akt pro-survival kinases. We now provide experimental evidence that DHEA activates these kinases via TrkA receptors. Down-regulation of TrkA receptors using siRNAs resulted in an almost complete reversal of the ability of DHEA to increase the phosphorylation of kinases Shc, Akt, and ERK1/2. In addition to TrkA receptors, binding of DHEA to the low affinity NGF receptor was also functional, affording the activation of p75^NTR^ receptors. Unlike TrkA receptors, p75^NTR^ lacks any enzymatic activity. Signal transduction by p75^NTR^ proceeds via ligand-dependent recruitment and release of cytoplasmic effectors to and from the receptor. Indeed, DHEA like NGF facilitated the recruitment of two major cytoplasmic interactors of p75^NTR^, TRAF6 and RIP2 proteins. Additionally, DHEA-mediated activation of p75^NTR^ led to the dissociation of bound RhoGDI, a protein belonging to small GTPases and interacting with RhoA [21]. A schematic representation of our findings is shown in Figure 7.

Previous findings suggest that DHEA protects PC12 cells against apoptosis via pertussis toxin (PTX) sensitive, G protein-associated specific plasma membrane-binding sites [9]. Indeed, PTX was shown to partially reverse the anti-apoptotic effects of DHEA and its membrane impermeable DHEA-BSA conjugate, as well as their effects on prosurvival kinases PI3K/Akt, the activation of transcription factor NFkappaB, and the phosphorylation and inactivation of apoptotic protein Bad [10]. Interestingly, the prosurvival effects of NGF in sympathetic neurons and PC12 cells are also partially reversed by PTX [38]. Furthermore, the NGF-dependent activation of Akt is partially attenuated by PTX, indicating the participation of G(i/o) proteins. In the same study, NGF-induced phosphorylation of Bad and transcriptional activity of NFkappaB were also shown to be sensitive to PTX [38]. It appears that other NGF-driven pathways are sensitive to PTX too. For instance, in PC12 cells and primary cortical neurons the NGF-induced phosphorylation of tuberin (a critical translation regulator holding a central role in NGF-promoted neuronal survival) is partially blocked by PTX, suggesting the participation of G(i/o) proteins [39]. Finally, NGF-dependent activation of the p42/p44 mitogen-activated protein kinase (p42/p44 MAPK) pathway in PC12 cells was effectively blocked by PTX [40]. However in HEK293 cells transfected with TrkA receptors, PTX was unable to affect the induction of TrkA phosphorylation by NGF or DHEA (Figure S5). These findings considered together suggest that TrkA receptors may use down-stream G protein-coupled receptor pathways, after binding and activation by NGF or DHEA, to control neuronal cell survival.

It is worth noticing that the interaction of DHEA with the NGF system was first suggested 15 years ago by Compagnone et al., showing co-localized staining of CYP17, the rate limiting enzyme of DHEA biosynthesis, and NGF receptors in mouse embryonic DRGs [41]. About one-fifth of CYP17-immunopositive DRG neurons in the mouse were found to be also TrkA-immunopositive. Among the TrkA-expressing cells, about one-third also express CYP17, while p75^NTR^-expressing neurons represent only 13% of the cells in the DRG. Thus, about one-fifth of CYP17-immunopositive neurons may be able to respond to both DHEA and NGF stimulation, an observation compatible with our data, presented in Figure 6C. A recent report further supports the interaction of DHEA with NGF receptors. Indeed, DHEA was shown to act as a keratinocyte-deriving neurotrophic signal, mimicking NGF in promoting axonal outgrowth of NGF non-producing but TrkA positive sensory neurons, an effect blocked by TrkA inhibitor K252a [42].

CYP17 is expressed in invertebrate cephalochordata Amphioxus [43]. Amphioxus is also expressing TrkA receptor homologous AmphiTrk, which effectively transduces signals mediated by NGF [44]. Phylogenetic analysis of neurotrophins revealed that they emerged with the appearance of vertebrates (530–550 million years ago), when complexity of neural tissue increased [45]. Invertebrate cephalochordata like Amphioxus are positioned on the phylogenetic boundary with vertebrates (600 million years ago). It is thus tempting to hypothesize that DHEA contributed as one of the “prehistoric” neurotrophic factors in an ancestral, simpler structurally invertebrate nervous system [46]; then, when a strict tempospatial regulation of evolving nervous system of vertebrates was needed, peptidic neurotrophins emerged to afford rigorous and cell specific neurodevelopmental processes.

In conclusion, our findings suggest that DHEA and NGF cross-talk via their binding to NGF receptors to afford brain shaping and maintenance during development. During aging, the decline of both factors may leave the brain unprotected against neurotoxic challenges. This may also be the case in neurodegenerative conditions associated with lower production or action of both factors. DHEA analogs may represent lead molecules for designing non-endocrine, neuroprotective, and neurogenic micromolecular NGF receptor agonists.

## Materials and Methods

### si/shRNAs, Plasmids, and Antibodies

PC12 cells were transfected with specific si/shRNAs for blocking the expression of TrkA and/or p75^NTR^ receptors. More specifically, three siRNAs and two shRNAs for TrkA and p75^NTR^, respectively, were obtained. The sequences for TrkA siRNAs (Ambion) were: GCCUAACCAUCGUGAAGAG (siRNA ID 191894), GCAUCCAUCAUAAUAGCAA (siRNA ID 191895), and CCUGACGGAGCUCUAUGUG (siRNA ID 191893). Sequences for p75^NTR^ (Qiagen) were: GACCUAUCUGAGCUGAAA (Cat. No. SI00251090) and GCGUGACUUUCAGGGAAA (CatNo SI00251083).

Rat TrkA was expressed from the pHA vector backbone and rat p75^NTR^ was expressed from the pCDNA3 vector backbone (InVitrogen) using a full length coding sequence flanked by an N-terminal hemagglutinin (HA) epitope tag. Plasmids to express RIP2 [19] and RhoGDI [36] were myc-tagged, while TRAF6 [19] was FLAG-tagged, as previously described.

The origin of antibodies was as follows: Bcl-2 (Cat. No. C-2, sc-7382, Santa Cruz Biotechnology Inc.), phospho TrkA (Cat. No. 9141, Cell Signaling), TrkA (Cat. No. 2505, Cell Signaling, was used for Western Blotting and Cat. No. 06-574, Upstate, was used for immunostainings), p75^NTR^ (Cat. No. MAB365R, Millipore), c-myc (Cat. No. 9E10, sc-40, Santa Cruz Biotechnology Inc.), phospho ERK1/2 (Cat. No. 9106, Cell Signaling), Erk1/2 (Cat. No. 9102, Cell Signaling), phospho-Shc (Tyr239/240) Antibody (Cat. No. 2434, Cell Signaling), Shc (Cat. No. 2432, Cell Signaling), phospho-Akt (Ser473) (Cat. No. 9271, Cell Signaling), Akt (Cat. No. 9272, Cell Signaling), anti-FLAG (M2) mouse monoclonal (Cat. No. F1804, Sigma), pTyr (Cat. No. sc-508, Santa Cruz Biotechnology Inc.), active Caspase-3 (Cat. No. ab13847, Abcam), Tyrosine Hydroxylase (Cat. No. ab6211, Abcam), anti-rabbit-R-phycoerythrin conjugated (Cat. No. P9537, Sigma), anti-mouse-fluorescein conjugated (Cat. No. AP124F, Millipore), anti-rabbit Alexa Fluor 488 (Cat. No. A21206), anti-rabbit Alexa Fluor 546 (Cat. No. A10040), and GAPDH (Cat. No. 2118, Cell Signaling).

### Cell Cultures and Transfection

PC12 cells were obtained from LGC Promochem (LGC Standards GmbH, Germany) and nnr5 cells from Dr. C.F. Ibáñez (Karolinska Institute). Both cell types were grown in RPMI 1640 containing 2 mM L-glutamine, 15 mM HEPES, 100 units/ml penicillin, 0.1 mg/ml streptomycin, and 10% horse serum, 5% fetal calf serum (both charcoal-stripped for removing endogenous steroids) at 5% CO_2_ and 37°C. HEK-293 cells were obtained from LGC Promochem. Cells were grown in DMEM medium containing 10% fetal bovine serum (charcoal-stripped for removing endogenous steroids), 100 units/ml penicillin, and 0.1 mg/ml streptomycin, at 5% CO_2_ and 3°C. HEK-293 and PC12 cells were transfected with Lipofectamine 2000 (InVitrogen) according to manufacturer's instructions. Transfected cells were typically used on the 2nd day after transfection.

### Measurement of Apoptosis

PC12 cells were cultured in 12-well plates, and 24 h later they were transfected with the si/shRNAs for TrkA and/or p75^NTR^. Twenty-four hours later the medium was aspirated and replaced either with complete medium (serum supplemented) or serum free medium in the absence or the presence of DHEA or DHEA-BSA conjugate at 100 nM. Apoptosis was quantified 24 h later with annexin V-FITC and PI (BD Pharmingen) according to our protocol [8].

### [^3^H]-DHEA Binding Assays

#### Membrane preparation

HEK293 cells transfected with an empty vector (HEK293-Vector) or overexpressing p75^NTR^ (HEK293-p75) or TrkA (HEK293-TrkA), and PC12 cells wild type or shRNA knocked down for p75^NTR^ and TrkA, were cultured, collected by scraping on ice and washed twice with cold Phosphate Buffer Saline (PBS), pH 7.4. After centrifugation at 1,200 rpm, cells were homogenized in a 50 mM Tris-HCl buffer, pH 7.4 (at 4°C), containing freshly added protease inhibitors (1 mM PMSF and 1 µg/ml aprotinin). Crude membrane fractions were isolated by differential centrifugation at 2,500×g (10 min at 4°C, to remove unbroken cells and nuclei) and 102,000×g (1 h, at 4°C). Membranes were washed once with ice-cold 50 mM Tris–HCl buffer, pH 7.4, and re-suspended in the same buffer. Membranes were then briefly acidified with 50 mM glycine pH 3 for 3 min on ice to elute membrane adsorbed proteins, washed once, resuspended in PBS (pH 7.4) with protease inhibitors, at a concentration of 2 mg/ml, and used immediately for binding experiments.

#### Binding conditions

Fifty µl of membrane suspension (2 mg/ml) in triplicate were incubated with 10 µl of 1–30 nM [^3^H]-DHEA (Perkin Elmer, Boston, MA) in the presence or absence of 500-fold molar excess of DHEA, in PBS, pH 7.4 with protease inhibitors, in a final volume of 100 µl. Membranes were incubated for 30 min at 25°C or overnight at 4°C, on a rotating plate; then they were collected on GF/B filters, prewetted in 0.5% PEI solution at 4°C. Filters were washed three times with ice-cold PBS, dried, and counted in scintillation fluid (SigmaFluor, Sigma) in a scintillation counter (Perkin Elmer, Foster City, CA) with 60% efficiency for Tritium. For saturation curves, specific binding (Bound, B) was calculated as the difference of Total Binding − Non Specific Binding. K_Ds_ were calculated from B/F over B Scatchard plots. Non-specific binding was ranging from 30% to 50% of total binding shown in the micromolar concentrations of ligand. Relatively higher levels of non-specific binding have been typically reported in binding assays for specific membrane binding of various steroid hormones, due to the highly lipophilic nature of phospholipid containing cell membranes combined with the strong lipophilicity of steroids. For displacement experiments, a constant concentration of [^3^H]-DHEA (1 nM) was incubated with increasing concentrations of competitors (10^−12^–10^−6^ M), under the same conditions as for saturation binding.

### Fluorescence Microscopy

HEK293 cells were allowed to grow on gelatin-coated glass coverslips for 24 h in culture medium, and 24 h later they were transfected with the cDNAs for TrkA, and p75^NTR^ receptors or the vector alone. Staining was performed 48 h after transfection. Culture medium was aspirated and transfectants were washed twice with PBS buffer. Primary antibodies against TrkA (rabbit, Upstate, No. 06-574, diluted 1∶100) or p75^NTR^ (mouse monoclonal ab, MAB365R, Millipore, dilution 1∶500) were added for 30 min at 37°C. Secondary antibodies, anti-rabbit-R-phycoerythrin conjugated (Sigma, No. P9537), and anti-mouse-fluorescein conjugated (No. AP124F, Millipore) were added at 1∶100 dilution and transfectants were incubated for 30 min at 37°C; then they were washed three times with PBS and counterstained with Hoechst nuclear stain (Molecular Probes) for 5 min. Transfectants were also incubated with the DHEA-BSA-FITC or the BSA-FITC conjugates (10^−6^M) for 15 min at room temperature in the dark; then they were washed with serum free culture medium and incubated for another 15 min in serum free culture medium containing 4% BSA. Coverslips were mounted to slides with 90% glycerin and were observed with a confocal laser scanning microscope (Leica TCS-NT, Leica Microsystems GmbH, Heidelberg, Germany), mounted with a digital camera.

### Flow Cytometry

HEK293 cells were cultured in 12-well plates, and 24 h later they were transfected with the cDNAs for TrkA and/or p75^NTR^ receptors, or the vector alone. Staining was performed 48 h later. Transfectants (5×10^5^ cells) were pelleted and incubated with 20 µl of the primary antibodies against TrkA or p75^NTR^ receptors for 30 min over ice. Afterwards, transfectants were washed three times with PBS and 20 µl of the secondary antibodies, and anti-rabbit-R-phycoerythrin conjugated and anti-mouse-fluorescein conjugated were added, as described above. For DHEA-BSA-FITC binding on cells, 20 µl (100 nM) were added on the pelleted cells for 10 min at RT, and then they were washed with serum free culture medium and incubated for another 15 min in serum free culture medium containing 4% BSA. Transfectants were washed twice with PBS, resuspended in 500 µl of PBS, and were analyzed in a Beckton-Dickinson FACSArray apparatus and the CELLQuest software (Beckton-Dickinson, Franklin Lakes, NJ).

### Synthesis of Immobilised DHEA-7-CMO

NovaPEG amino resin (loading value 0.78 mmol/g) was purchased from Novabiochem. NMR spectra were recorded on a Varian 300 spectrometer operating at 300 MHz for ^1^H and 75.43 MHz for ^13^C or on a Varian 600 operating at 600 MHz for ^1^H. ^1^H NMR spectra are reported in units of δ relative to the internal standard of signals of the remaining protons of deuterated chloroform, at 7.24 ppm. ^13^C NMR shifts are expressed in units of *δ* relative to CDCl_3_ at 77.0 ppm. ^13^C NMR spectra were proton noise decoupled. IR spectra was recorded at Bruker Tensor 27. Absorption maxima are reported in wavenumbers (cm^−1^).

3*β*-Acetoxy-17,17-ethylenedioxyandrost-5-ene (0.74 g, 1.98 mmol) and N-hydroxy phthalimide (0.71 g, 2.2 mmol) were dissolved in acetone (39 mL) containing 1 mL of pyridine. The mixture was stirred vigorously at room temperature and sodium dichromate dihydrate (0.89 g, 3 mmol) was added. Additional portions of solid sodium dichromate dihydrate (0.89 g, 3 mmol) were added after 10 and 20 h stirring at room temperature. After reaction completion (48 h), the mixture was diluted with dichloromethane, filtered through a bed of celite, and the filtrate was washed with water, saturated sodium bicarbonate solution, and brine. The organic layer was dried over anhydrous sodium sulfate, the solvent evaporated *in vacuo*, and the residue purified by flash column chromatography using hexane/acetone/25% NH_4_OH (85∶15∶0.1 mL) as eluent to afford 3*β*-acetoxy-17,17-ethylenedioxyandrost-5-ene-7-one (0.6 g, yield: 78%). ^1^H NMR (CDCl_3_, 300 MHz) *δ*: 0.87 (s, 3H), 1.21 (s, 3H), 1.26–2.00 (m, 14H), 2.05 (s, 3H), 2.20–2.51 (m, 3H), 3.84–3.92 (m, 4H), 4.68–4.76 (m, 1H), 5.70 (d, *J* = 1.58 Hz, 1H).

To a solution of 3*β*-acetoxy-17,17-ethylenedioxyandrost-5-en-7-one (0.1 g, 0.26 mmol) in pyridine (1.9 mL) was added O-(carboxymethyl)hydroxylamine hemihydrochloride (0.11 g, 0.52 mmol) and the reaction mixture was stirred overnight under argon. After completion of the reaction, the solvent was evaporated and the residue was diluted with ethyl acetate. The organic layer was washed with water and brine, dried over anhydrous sodium sulfate, and the solvent was evaporated *in vacuo* to afford 3*β*-acetoxy-17,17-ethylenedioxyandrost-5-en-7-one7-(O-carboxymethy1) oxime as a white foam (0.12 g, yield: 100%). ^1^H NMR (CDCl_3_, 300 MHz) *δ*: 0.88 (s, 3H), 1.13 (s, 3H), 1.16–1.95 (m, 12H), 2.04 (s, 3H), 2.25–2.59 (m, 5H), 3.84–3.95 (m, 4H), 4.59 (d, *J* = 2.29 Hz, 2H), 4.62–4.73 (m, 1H), 6.51 (d, *J* = 1.47 Hz, 1H).

To a solution of 3*β*-acetoxy-17,17-ethylenedioxyandrost-5-en-7-one-7-(O-carboxymethy1) oxime (0.12 g, 0.26 mmol) in a mixture of acetone/water (5∶1, 6.3 mL) was added *p*-toluenesulfonic acid monohydrate (0.019 g, 0.10 mmol), and the reaction mixture was stirred until the starting material was consumed (48 h). The solvent was evaporated *in vacuo* and the residue was diluted with ethyl acetate. The organic layer was washed with water and brine, dried over anhydrous sodium sulfate, and the solvent was evaporated *in vacuo* to afford 3β-acetoxy-androst-5-en-7,17-dione 7-(O-carboxymethy1) oxime as a white foam (0.11 g, yield: 100%). ^1^H NMR (CDCl_3_, 600 MHz) *δ*: 0.90 (s, 3H), 1.15 (s, 3H), 1.20–1.95 (m, 12H), 2.05 (s, 3H), 2.09–2.68 (m, 5H), 4.63 (d, *J* = 4.18 Hz, 2H), 4.65–4.71 (m, 1H), 6.56 (d, *J* = 1.39 Hz, 1H).

To a solution of 3β-acetoxy-androst-5-en-7,17-dione 7-(O-carboxymethy1) oxime (0.11 g, 0.26 mmol) in methanol (3.9 mL) was added LiOH (1.5 mL, 1.5 mmol, 1N solution), and the reaction mixture was stirred until the starting material was consumed (4 h). The solvent was evaporated *in vacuo* and the residue was diluted with water. The solution was acidified with 10% hydrochloric acid and DHEA-7-CMO precipitated as a white solid, which was isolated by filtration (0.097 g, yield: 100%). ^1^H NMR (CDCl_3_/CD_3_OD, 600 MHz) *δ*: 0.90 (s, 3H), 1.14 (s, 3H), 1.20–2.75 (m, 17H), 3.49–3.54 (m, 1H), 4.54 (s, 2H), 6.54 (s, 1H).

3*β*-Hydroxy-17-oxoandrost-5-en-7-*O*-(carboxymethyl)oxime (DHEA-7-CMO) (192 mg, 0.511 mmol) in DMF (5 mL) was treated with HOBt (69 mg, 0.511 mmol) and DIC (0.08 mL, 0.511 mmol), and the resulting mixture was stirred at room temperature for 30 min. This solution was added to NovaPEG amino resin (130 mg, 0.102 mmol, 0.78 mmol/gr) (pre-swollen with DMF for 1 h) and the slurry was shaken at room temperature overnight. The mixture was filtered, the resin was sequentially washed with dichloromethane (3×), methanol (3×), and diethyl ether (3×), and was dried *in vacuo* overnight. Yield 175 mg (100%), loading value 0.61 mmol/gr. ^13^C NMR (gel phase, CDCl_3_) *δ*: 220.66, 170.15, 157.10, 154.15, 113.11, 72.57, 66.59, 49.92, 47.86, 42.15, 38.46, 37.08, 36.53, 35.49, 31.20, 30.71, 24.96, 20.15, 18.05, 13.95; IR: *ν*_max_/cm^−1^ 2865 (s), 1735 (m), 1669 (w), 1653 (w), 1637 (w), 1456 (m), 1348 (w), 1289 (w), 1247 (w), 1093 (s), 946 (w).

### Co-immunoprecipitation and Pull-Down Assays

HEK293 cells were transfected with the appropriate plasmids (TrkA, p75^NTR^, RIP2, TRAF-6, and RhoGDI) by using Lipofectamine 2000 (Invitrogen). Cells were harvested 48 h after transfection and suspended in lysis buffer (50 mM Tris-HCl, 0.15 M NaCl, 1% Triton-X100, pH 7.4) supplemented with protease inhibitors. Lysates were precleared for 1 h with Protein A-Sepharose beads (Amersham) and immunoprecipitated with the appropriate antibody (pTyr, Flag, or c-myc) overnight at 4°C. Protein A Sepharose beads were incubated with the lysates for 4 h at 4°C with gentle shaking. In the case of immobilized DHEA-7-CMO, HEK293 or PC12 cells lysates or purified receptors (both from R&D Systems, Recombinant Mouse NGF R/TNFRSF16/Fc Chimera, Cat. No.: 1157-NR and Recombinant Rat Trk A/Fc Chimera, Cat. No.: 1056-TK) were incubated overnight at 4°C with the NovaPEG amino resin alone or conjugated with DHEA. Beads were collected by centrifugation, washed four times with lysis buffer, and resuspended in SDS loading buffer. Proteins were separated by SDS/PAGE, followed by immunoblotting with specific antibodies.

### Western Blot Analysis

PC12 or HEK293 cells lysates were electrophoresed through a 12% SDS-polyacrylamide gel, and then proteins were transferred to nitrocellulose membranes, which were processed according to standard Western blotting procedures, as previously described [8]. To detect protein levels, membranes were incubated with the appropriate antibodies: Bcl-2 (dilution 1∶500), phospho TrkA (dilution 1∶500), total TrkA (dilution1∶500), p75^NTR^ (dilution 1∶500), phospho Shc (dilution 1∶1000), total Shc (dilution 1∶1000), phospho Akt (dilution 1∶500), total Akt (dilution 1∶500), phospho ERK1/2 (dilution 1∶500), and total ERK1/2 (dilution 1∶500). Proteins were visualized using the ECL Western blotting kit (ECL Amersham Biosciences, UK), and blots were exposed to Kodak X-Omat AR films. A PC-based Image Analysis program was used to quantify the intensity of each band (Image Analysis, Inc., Ontario, Canada).

To normalize for protein content the blots were stripped and stained with GAPDH antibody (dilution 1∶1000); the concentration of each target protein was normalized versus GAPDH. Where phosphorylation of TrkA or kinases was measured, membranes were first probed for the phosphorylated form of the protein, then stripped, and probed for the total protein.

### Superior Cervical Ganglia Neuronal Cultures

Superior cervical ganglia (SCG) were removed from newborn (P0–P1) rat pups and dissociated in 0.25% trypsin (Gibco, 15090) for 30 min at 37°C. After dissociation SCG neurons were re-suspended in culture medium (Gibco, Neurobasal Cat. No. 21103) containing 1% fetal bovine serum (FBS), 100 units/ml penicillin, 0.1 mg/ml streptomycin, 3 µg/ml araC antimitotic, and 100 ng/ml NGF (Millipore, 01-125). Cells were plated on collagen coated 24-well plates and cultured for 5 d prior to use. For NGF withdrawal experiments, cells were washed twice with Neurobasal containing 1% FBS and fresh culture medium lacking NGF and containing anti-NGF antibody at 1∶50 dilution (Millipore, AB1526). DHEA, TrkA-inhibitor (Calbiochem, 648450) and anti-p75^NTR^ (mouse, MAB365R Millipore) were used at 100 nM, 100 nM, and 1∶50, respectively.

### In Vivo Experiments with the NGF Null Mice

*ngf*+/− mice [13] were obtained from the Jackson Laboratory and maintained on C57BL/6 background. All procedures described below were approved by the Animal Care Committee of the University of Crete, School of Medicine. Animals were housed in cages maintained under a constant 12 h light–dark cycle at 21–23°C, with free access to food and tap water. Genotyping was performed on tail DNA using the following primers: NGFKOU2 (5′CCG TGA TAT TGC TGA AGA GC3′), NGFU6 (5′CAG AAC CGT ACA CAG ATA GC3′), and NGFD1 (5′TGT GTC TAT CCG GAT GAA CC3′). Genomic PCR reactions containing the 3 primers were incubated for 32 cycles at 95°C (30 s)/59°C (30 s)/72°C (1 min).

Mice heterozygous for the NGF null mutation were interbred to obtain mice homozygous for the NGF gene disruption and the first day of gestation determined by the discovery of a copulation plug. The mothers were treated daily with a subcutaneous injection of DHEA (2 mg/day) or vehicle (4.5% ethanol in 0.9% saline) starting from the third day after gestation. Animals were collected at E14. At the day of collection the mothers were deeply anesthetized with sodium pentobarbital (Dolethal 0.7 ml/kg i.p.) followed by transcardial perfusion with saline solution containing heparin for about 7 min, and with 4% PFA, 15% Picric Acid, 0.05% GA in phosphate buffer 0.1 M, for another 7 min. After the perfusion the embryos were collected and maintained in the same fixative overnight at 4°C. Embryos were then washed in 0.1 M phosphate buffer and cryoprotected by using 10% sucrose followed by 20% sucrose overnight at 4°C. Finally, embryos were frozen in OCT in iso-pentane over liquid nitrogen for 5 min and the frozen tissues were stored for later use at −80°C. The samples were sectioned (20 µm) and mounted onto Superfrost plus slides (Menzel-Glaser J1800AMNZ). Slides were left to air-dry overnight at room temperature (RT) and were then either used immediately or were fixed in cold acetone for 1 min and stored at −80°C for later use.

Stored or fresh slides were fixed for 15 min in cold acetone at 4°C and left to dry for 10 min at room temperature. They were then washed in PB 0.1 M, then in TBS, and incubated for 45 min with 10% horse serum in TBS-T 0.1%. The normal serum was drained off and the primary antibodies (anti-TrkA diluted 1∶400 and active Caspase-3 diluted 1∶50), diluted in TBS-T 0.1% with 1% horse serum, were added. Sections were incubated for 4 h at RT and overnight at 4°C; they were then washed in TBS-T 0.1% and the anti-rabbit secondary antibodies (Alexa Fluor 488 and Alexa Fluor 546, 1∶1000 in TBS-T 0.1%) were added for 6 h at RT. Sections were washed in TBS-T, TBS, and in PB 0.1 M and were coverslipped with Vectashield (Vector, H-1400) and visualized in a confocal microscope. TUNEL (Roche, Cat. No. 12156792910) and Fluoro-Jade C (Millipore, Cat. No. AG325) staining of apoptotic and degenerating neurons, respectively, was performed according to the manufacturer's instructions.

### Statistical Analysis

For the statistical evaluation of our data we have used analysis of variance, post hoc comparison of means, followed by the Fisher's least significance difference test. For data expressed as percent changes we have used the nonparametric Kruskal-Wallis test for several independent samples.

## Supporting Information

Figure S1 DHEA regulates the levels of TrkA and p75^NTR^ receptors, mimicking NGF. Serum deprived PC12 cells were exposed to 100 nM of DHEA or 100 ng/ml of NGF for 12, 14, and 48 h. TrkA and p75^NTR^ protein levels were measured in cell lysates with immunoblotting, using specific antibodies, and were normalized against GAPDH (* *p*<0.01 versus control-Serum Free, *n* = 5).
(TIF)

Figure S2 [^3^H]-DHEA saturation binding assays and Scatchard blots in HEK293 cells, transfected with the plasmid cDNAs of TrkA and p75^NTR^ receptors. Fifty µl of cell membrane suspension in triplicate were incubated overnight at 4°C with 1–30 nM [^3^H]-DHEA in the presence or absence of 500-fold molar excess of DHEA.
(TIF)

Figure S3 RT-PCR of androgen receptors (AR) mRNA in HEK23 cells, transfected with the plasmid cDNAs of TrkA and p75^NTR^ receptors. Total RNA was extracted from LNCaP and naïve HEK293 cells or HEK293 cells transfected with the plasmid cDNAS of TrkA, p75^NTR^, or both plasmids, by using the Trizol Reagent (InVitrogen). One microgram of total RNA was reverse transcribed by using the Thermo-Script RT-PCR System (InVitrogen). The cDNA was amplified by PCR. PCR was performed in a Perkin–Elmer DNA Thermal Cycler with the following conditions: 60 s at 94°C, 60 s at 60°C, and 3 min at 72°C (for Androgen Receptor, AR) or 30 s at 94°C, 30 s at 58°C, and 60 s at 72°C (for GAPDH), for 30 cycles to detect the product at the exponential phase of the amplification. Ten microliters of the amplified products (1.4 Kb for AR and 483 bp for GAPDH) were separated on a 1.5% agarose gel and visualized by ethidium bromide staining. Primers for AR were 5′-AGCTACTCCGGACCTTACG-3′ (sense) and 5′-AGGTGCCATGGGAGGGTTAG-3′ (antisense) and primers for GAPDH were 5′-GCCACATCGCTCAGACACCA-3′ (sense) and 5′-GATGACCCTTTTGGCTCCCC-3′ (antisense).
(TIF)

Figure S4 Representative curves of [^3^H]-DHEA displacement experiments. The selectivity of DHEA binding to HEK293^TrkA^ and HEK293^p75NTR^ cell membranes was examined by performing heterologous [^3^H]-DHEA displacement experiments using a number of non-labeled steroids or NGF. Membranes (at a final concentration of 2 mg protein/ml) isolated from HEK293 cells transfected with the plasmid cDNAs of TrkA and p75^NTR^ receptors were incubated with 1 nM [^3^H]-DHEA in the absence or the presence of various unlabeled steroids or NGF at concentrations varying from 0.01 to 1,000 nM. Binding of [^3^H]-DHEA to membranes isolated from both HEK293^TrkA^ and HEK293^p75NTR^ cells was effectively displaced by NGF (IC_50_: 0.8±0.2 and 1.19±0.45 nM, respectively, *n* = 4). Estradiol failed to displace [^3^H]-DHEA from its binding on membranes from HEK293^TrkA^ and HEK293^p75NTR^ cells at concentrations ranging from 0.1 to 1,000 nM. In contrast, displacement of [^3^H]-DHEA binding to membranes from both HEK293^TrkA^ and HEK293^p75NTR^ cells was shown by sulfated ester of DHEA, DHEAS (IC_50_: 6.1±1.1 and 8.1±1.2 nM, respectively, *n* = 3), and testosterone (Testo) (IC_50_: 5.3±2.1 and 7.4±3.2 nM, respectively, *n* = 4). Glucocorticoid dexamethasone (DEX) effectively competed [^3^H]-DHEA binding to membranes from HEK293^TrkA^ (IC_50_: 9.5±4.6 nM, *n* = 4) but was ineffective in displacing DHEA binding to membranes from HEK293^p75NTR^ cells.
(TIF)

Figure S5 Effects of PTX, DHEAS, and testosterone on TrkA phosphorylation in HEK293^TrkA^ cells. HEK293^TrkA^ transfectants cultured in serum free conditions were exposed for 10 min to 100 nM of DHEA or 100 ng/ml of NGF, in the absence or the presence of 100 ng/ml PTX, or to 100 nM of DHEAS or testosterone. Cell lysates were then immunoprecipitated overnight at 4°C with anti-tyrosine antibody and analyzed by Western blotting, using specific antibodies against TrkA receptors.
(TIF)

## Abbreviations

BDNF: brain-derived neurotrophic factor
CYP17: cytochrome P450 17-hydroxylase/17,20-lyase
DHEA: dehydroepiandrosterone
DHEA-BSA: DHEA–Bovine Serum Albumin
DHEA-BSA-FITC: DHEA-BSA–Fluorescein Isothiocyanate
DHEA-PEG: DHEA–polyethylene glycol amino resin
DHEAS: dehydroepiandrosterone sulfate
DRG: dorsal root ganglia
GAPDH: glyceraldehyde-3-phosphate dehydrogenase
HEK293: human embryonic kidney cell line 293
MPTP: 1-methyl-4-phenyl-1,2,3,6-tetrahydropyridine
NGF: nerve growth factor
p75^NTR^: p75 neurotrophin receptor
PTX: pertussis toxin
RhoGDI: rho GDP dissociation inhibitor
RIP2: receptor-interacting protein 2
SCG: superior cervical ganglia
TRAF6: TNF receptor-associated factor 6
Trk: tropomyosin related kinase
