# Supplementary figures and images for: Neurosteroid Dehydroepiandrosterone Interacts with Nerve Growth Factor (NGF) Receptors, Preventing Neuronal Apoptosis
Source: PLoS Biol. 2011 Apr 26;9(4):e1001051. doi: 10.1371/journal.pbio.1001051 (PMC3082517; doi:10.1371/journal.pbio.1001051)

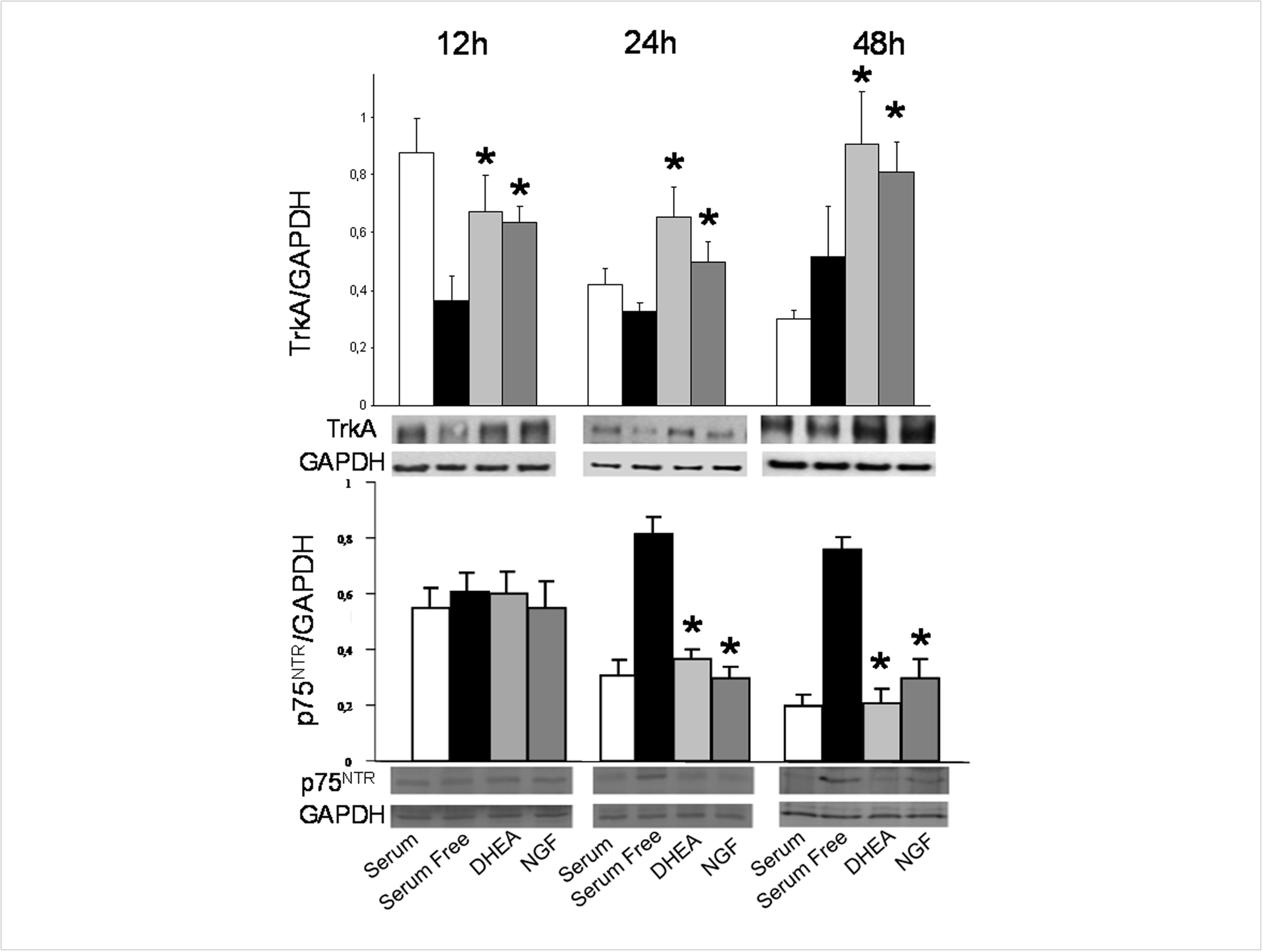

Supplement: Figure S1 — DHEA regulates the levels of TrkA and p75NTR receptors, mimicking NGF. Serum deprived PC12 cells were exposed to 100 nM of DHEA or 100 ng/ml of NGF for 12, 14, and 48 h. TrkA and p75NTR protein levels were measured in cell lysates with immunoblotting, using specific antibodies, and were normalized against GAPDH (* p<0.01 versus control-Serum Free, n = 5). (TIF) [file pbio.1001051.s001.tif]

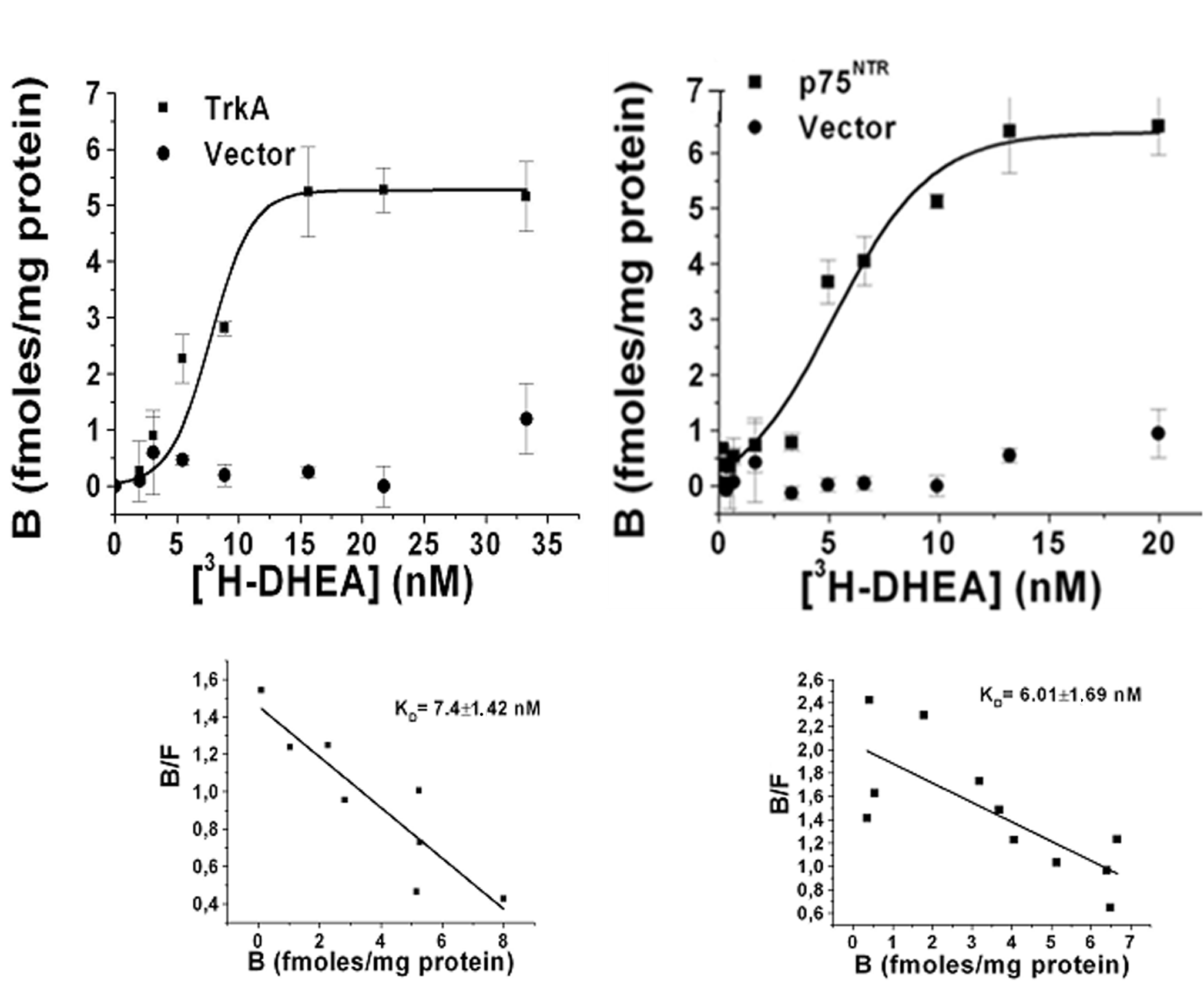

Supplement: Figure S2 — [3H]-DHEA saturation binding assays and Scatchard blots in HEK293 cells, transfected with the plasmid cDNAs of TrkA and p75NTR receptors. Fifty µl of cell membrane suspension in triplicate were incubated overnight at 4°C with 1–30 nM [3H]-DHEA in the presence or absence of 500-fold molar excess of DHEA. (TIF) [file pbio.1001051.s002.tif]

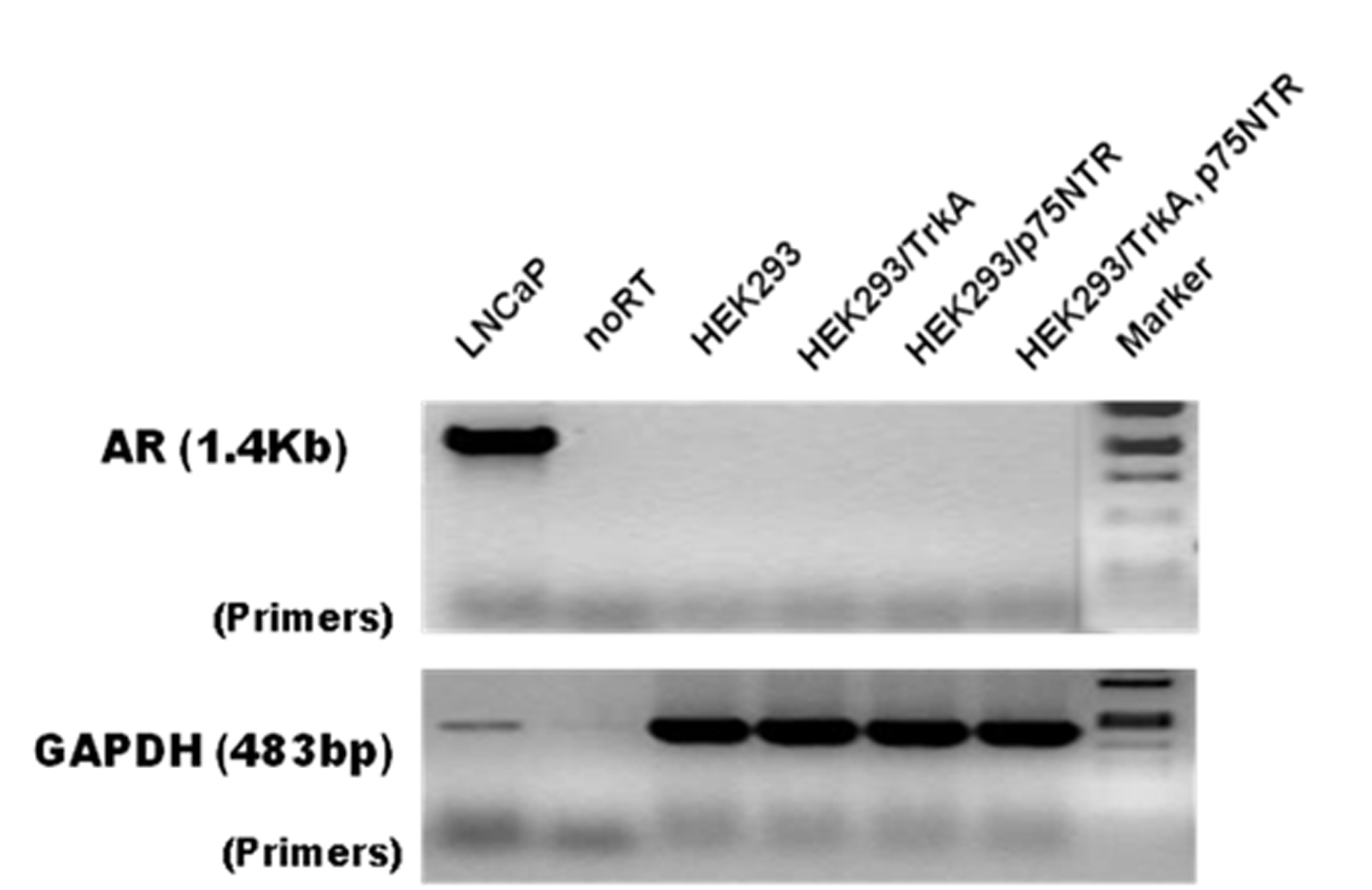

Supplement: Figure S3 — RT-PCR of androgen receptors (AR) mRNA in HEK23 cells, transfected with the plasmid cDNAs of TrkA and p75NTR receptors. Total RNA was extracted from LNCaP and naïve HEK293 cells or HEK293 cells transfected with the plasmid cDNAS of TrkA, p75NTR, or both plasmids, by using the Trizol Reagent (InVitrogen). One microgram of total RNA was reverse transcribed by using the Thermo-Script RT-PCR System (InVitrogen). The cDNA was amplified by PCR. PCR was performed in a Perkin–Elmer DNA Thermal Cycler with the following conditions: 60 s at 94°C, 60 s at 60°C, and 3 min at 72°C (for Androgen Receptor, AR) or 30 s at 94°C, 30 s at 58°C, and 60 s at 72°C (for GAPDH), for 30 cycles to detect the product at the exponential phase of the amplification. Ten microliters of the amplified products (1.4 Kb for AR and 483 bp for GAPDH) were separated on a 1.5% agarose gel and visualized by ethidium bromide staining. Primers for AR were 5′-AGCTACTCCGGACCTTACG-3′ (sense) and 5′-AGGTGCCATGGGAGGGTTAG-3′ (antisense) and primers for GAPDH were 5′-GCCACATCGCTCAGACACCA-3′ (sense) and 5′-GATGACCCTTTTGGCTCCCC-3′ (antisense). (TIF) [file pbio.1001051.s003.tif]

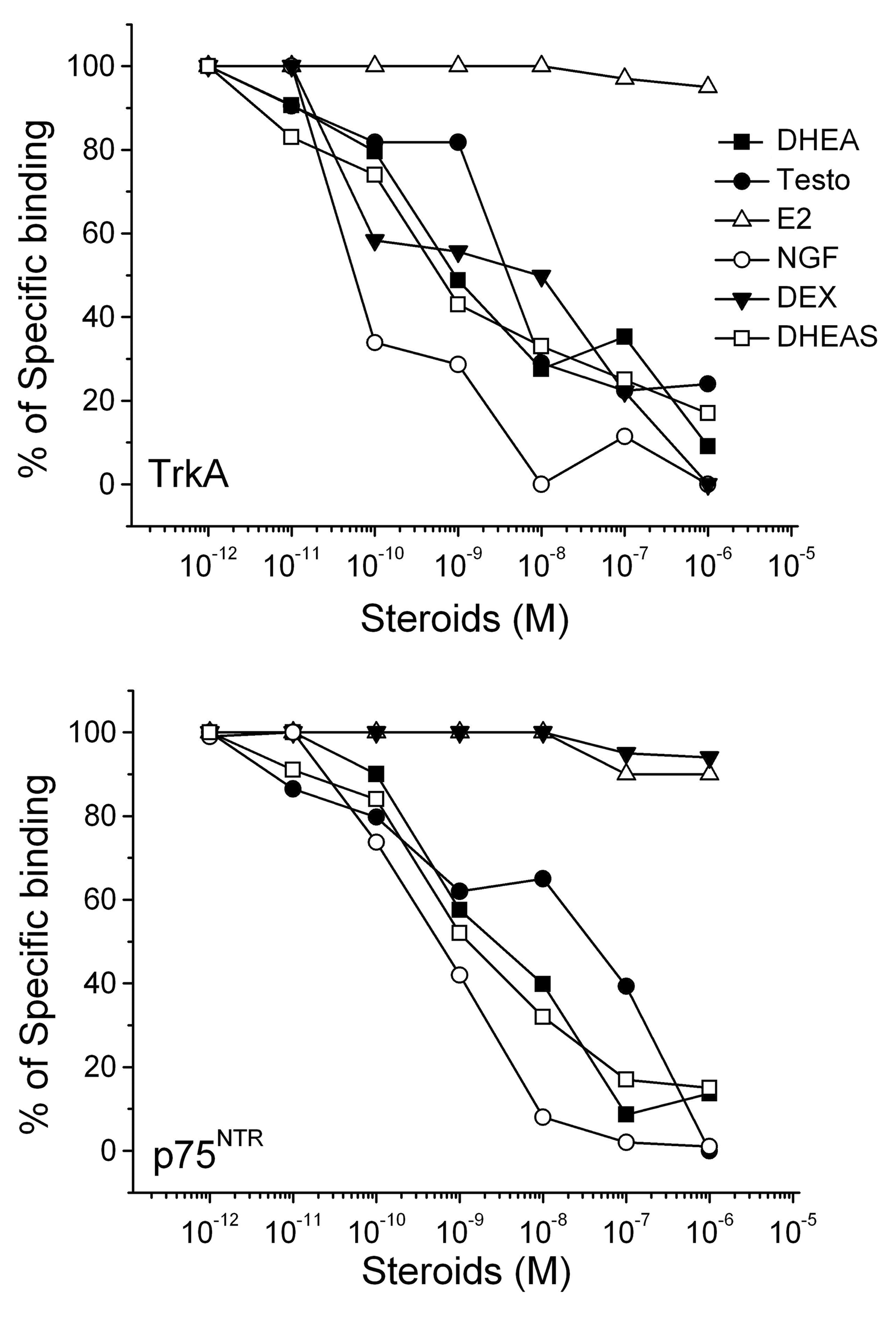

Supplement: Figure S4 — Representative curves of [3H]-DHEA displacement experiments. The selectivity of DHEA binding to HEK293TrkA and HEK293p75NTR cell membranes was examined by performing heterologous [3H]-DHEA displacement experiments using a number of non-labeled steroids or NGF. Membranes (at a final concentration of 2 mg protein/ml) isolated from HEK293 cells transfected with the plasmid cDNAs of TrkA and p75NTR receptors were incubated with 1 nM [3H]-DHEA in the absence or the presence of various unlabeled steroids or NGF at concentrations varying from 0.01 to 1,000 nM. Binding of [3H]-DHEA to membranes isolated from both HEK293TrkA and HEK293p75NTR cells was effectively displaced by NGF (IC50: 0.8±0.2 and 1.19±0.45 nM, respectively, n = 4). Estradiol failed to displace [3H]-DHEA from its binding on membranes from HEK293TrkA and HEK293p75NTR cells at concentrations ranging from 0.1 to 1,000 nM. In contrast, displacement of [3H]-DHEA binding to membranes from both HEK293TrkA and HEK293p75NTR cells was shown by sulfated ester of DHEA, DHEAS (IC50: 6.1±1.1 and 8.1±1.2 nM, respectively, n = 3), and testosterone (Testo) (IC50: 5.3±2.1 and 7.4±3.2 nM, respectively, n = 4). Glucocorticoid dexamethasone (DEX) effectively competed [3H]-DHEA binding to membranes from HEK293TrkA (IC50: 9.5±4.6 nM, n = 4) but was ineffective in displacing DHEA binding to membranes from HEK293p75NTR cells. (TIF) [file pbio.1001051.s004.tif]

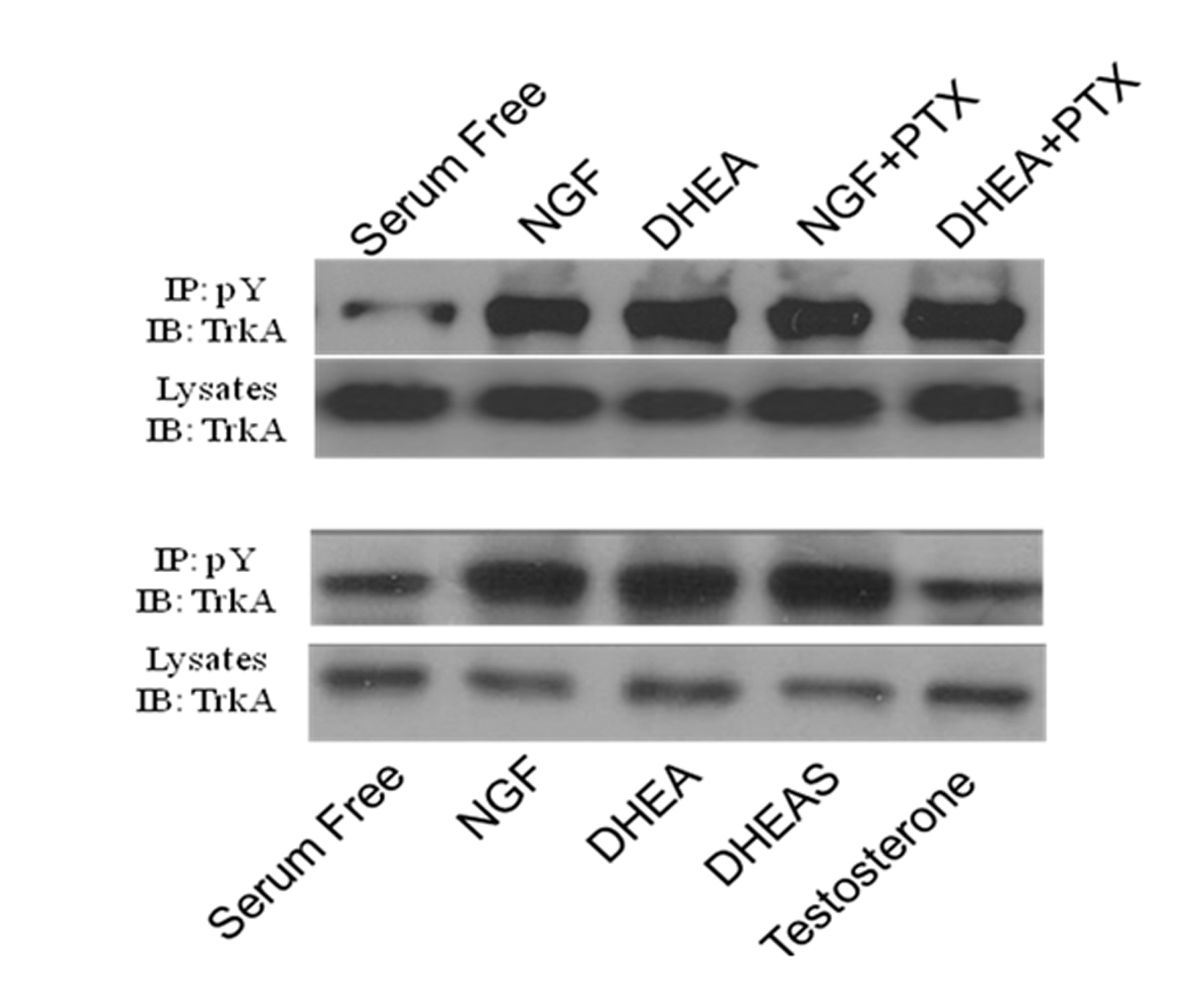

Supplement: Figure S5 — Effects of PTX, DHEAS, and testosterone on TrkA phosphorylation in HEK293TrkA cells. HEK293TrkA transfectants cultured in serum free conditions were exposed for 10 min to 100 nM of DHEA or 100 ng/ml of NGF, in the absence or the presence of 100 ng/ml PTX, or to 100 nM of DHEAS or testosterone. Cell lysates were then immunoprecipitated overnight at 4°C with anti-tyrosine antibody and analyzed by Western blotting, using specific antibodies against TrkA receptors. (TIF) [file pbio.1001051.s005.tif]
